# Age, gender, and score distributions of moral foundations

**DOI:** 10.1371/journal.pone.0352584

**Published:** 2026-07-01

**Authors:** Michael Zakharin, Timothy C. Bates

**Affiliations:** Department of Psychology, University of Edinburgh, Edinburgh, Scotland; Toulouse Business School: TBS Education, SPAIN

## Abstract

Claims of generational differences in moral values have important implications for understanding societal change. Using the recently developed Moral Foundations Questionnaire (MFQ-2), we examined age and gender effects on moral foundations in large US (N = 835) and UK (N = 1,659) samples. Across both nations, binding foundations (proportionality, loyalty, authority and purity) showed consistent age-related increases. While these yearly increments were modest, they accumulated to substantial differences between youngest and oldest participants, with binding scores approximately 0.75 standard deviations higher in older adults. Among individualizing foundations, the preference for equality decreased with age, while harm/care remained stable. Gender differences emerged systematically: women scored higher on individualizing foundations, while men showed elevated scores on binding foundations. We also observed significant distributional effects, with care scores clustering near the scale maximum and purity scores near the minimum. These demographic patterns suggest important dynamics in how moral values may shift across generations and between genders, with implications for understanding social change.

## Introduction

“*Children began to be the tyrants, not the slaves, of their households. They no longer rose from their seats when an elder entered the room; they contradicted their parents, chattered before company, gobbled up the dainties at table, and committed various offences against Hellenic tastes, such as crossing their legs. They tyrannised over the paidagogoi and schoolmasters*” [1].

As noted by Freeman [1], ancient writers, particularly those from ancient Greece such as Aristophanes and Xenophon, argued that the young of their day lacked the moral rectitude they themselves had shown at their age. These persistent claims of generational moral differences warrant rigorous investigation, as documenting either the presence or absence of age-related variations in moral foundations carries significant implications for understanding societal changes. Differences between young and old, of course, may refer to very different phenomena and causes [2]. One possibility is that moral foundations exhibit reliable developmental change, as suggested by classic theories of moral development, which propose a stage-like process of increasing moral concern [3,4]. Alternatively, change may affect the culture as a whole, impacting persons of all ages and manifesting as a shift in the mean score for the population. In such mean-shift cases, older generations who lived through an earlier, perhaps stricter (or more lax) time will observe that young people differ not only from their own youth but potentially from how their parents were at the same age. Here, we use the Moral Foundations Questionnaire- 2 (MFQ-2) to examine differences in the moral foundations in two new samples, one US and one UK, to describe mean differences in moral foundations by age and gender and the shape of MFQ-2 scores in the population—specifically examining distributional properties such as skew and ceiling/floor effects—and the changes exhibited with age. We first provide background on moral foundation theory and work on age and gender differences in foundation scores.

Moral foundations theory [MFT; 5–7] proposes that moral judgment is supported by multiple partially distinct concerns rather than a single moral principle. In this paper we focus on the six foundations assessed by the MFQ-2: care, equality, proportionality, loyalty, authority, and purity. Care captures concern for others’ suffering and harm prevention. Equality reflects egalitarian concern that people should be treated similarly and have similar outcomes. Proportionality reflects merit-based fairness – benefits and punishments should track effort, contribution, or responsibility, conceptually related to mutualism [8,9]. Loyalty concerns commitment to one’s group and aversion to betrayal. Authority concerns respect for legitimate hierarchy, tradition, and role-based duties. Purity concerns contamination and sanctity, including bodily and spiritual cleanliness and self-control. These foundations are often summarized into higher-order clusters in Western samples, with individualizing concerns (care, equality) emphasizing protection and equal regard for individuals, and binding concerns (loyalty, authority, purity, and proportionality) emphasizing group cohesion, order, and norm enforcement [6,7].

Moral foundations are commonly measured with the MFQ-1 [6] and the updated MFQ-2 [7]. The key conceptual difference is that MFQ-1 assesses five foundations, including a single fairness scale, whereas recently developed MFQ-2 assesses six foundations by splitting fairness into two separable constructs: equality and proportionality. In addition, MFQ-2 replaces the original MFQ-1 items with newly written or rephrased items, intended to improve clarity and content coverage and to better recover the expected factor structure in contemporary samples [7,10], addressing well-documented psychometric limitations of MFQ-1 in some datasets [e.g., 11,12–14]. The complete item sets for MFQ-1 and MFQ-2 are provided in the Supporting Information.

Having described the MFT foundations and measures, we next turn to existing research on the relationships of age and sex to moral foundations, focussing on studies with large sample sizes that offer the most relevant and robust findings.

### Age differences in moral foundations scores

The most comprehensive examination of age effects on moral foundations scores is the meta-analysis of 239 independent samples and 492,298 subjects using the MFQ-1, as reported by Castilla-Estévez and Blázquez-Rincón [15]. Their analysis revealed a consistent albeit small (mean r = 0.06) age-related increase in moral concern across all foundations (see Table 1). Two studies not included in [15] support this finding, with Nilsson [16] replicating the increase in all foundations in a Swedish sample (N = 2,282), with around twice the effect size as reported by Castilla-Estévez and Blázquez-Rincón [15]. In a New Zealand sample, Milojev, Osborne [17] also found that scores on all foundations increased with age, with a mean age effect of  .21 and larger effects in binding than individualizing. These studies and their estimates of age effects on moral foundations are summarized in Table 1. While these effects may appear modest, it is important to note that small increases, when accumulated over many decades of life, result in substantial differences in scores between the youngest and oldest members of the population.

**Table 1:** Correlations between moral foundations and age across the previous studies.

	Individualizing		Binding			
Study	Care	Fairness/Equality	Proportionality	Loyalty	Authority	Purity
Castilla-Estévez & Blázquez-Rincón (2021) N = 492,298	.08	.06	--	.04	.06	.07
Nilsson (2023) N = 2,282	.13	.07	--	.18	.07	.13
Milojev et al. (2014) N = 3635	.05	.15	--	.26	.29	.29
Atari et al. (2023; MFQ-2) N = 1410	.09	−.08	.04	.11	.12	.09

### Gender differences in moral foundations scores

The most extensive study of gender differences in MFQ-1 scores comes from Study 1 of Atari, Lai and Dehghani (18), reporting data from the online international dataset hosted on YourMorals.org (N = 348,660, with 67 countries represented with the majority (N = 262,629) coming from the US). Atari, Lai and Dehghani [18] reported higher scores in women for care, fairness, and purity, with care showing the greatest disparity. However, no significant differences were observed for loyalty or authority. These patterns were confirmed in an independent international sample (N = 11,969), although the gender gap in care scores was narrower, more closely matching the differences observed in fairness and purity (see Table 2). Similar results were also reported by Van Leeuwen, Dukes [19] using an international sample primarily from Western countries (N = 2,478), see Table 2. Again, women scored higher on care, fairness, and purity, and men scored higher on loyalty, with no difference found in authority. Likewise, in a Swedish sample (N = 2,282), Nilsson [16] replicated gender differences in care and fairness alongside a slight male advantage in authority, though the gender effect on purity did not hold. Finally, a study from New Zealand (N = 3,635) by Milojev, Osborne [17] echoed the gender discrepancies in care and fairness and found modest but significant gender differences in the binding foundations favouring men, with no significant effects found for purity.

**Table 2:** Gender differences in MFQ-1 moral foundations across the previous studies.

		Foundation					
Study	N	Care	Fairness	Proportionality	Loyalty	Authority	Purity
Milojev et al. (2014)	3,635	.25	.07		−.07	−.09	−.02
Atari et al. (2020), study 1	348,660	.45	.17		.03	.00	.20
Atari et al. (2020), study 2	11,969	.27	.15		−.05	−.03	.11
Nilsson (2023)	2,282	.21	.11		−.03	−.06	.02
Van Leeuwen et al. (2017)	2,478	.20	.08		−.04	.00	.10
Atari et al. (2023; MFQ-2)	3,902	.03	.16	−.09	−.06	−.06	.09
**Average**		.23	.12	−.09	−.04	−.04	.08

*Note*: Positive values indicate higher scores for women. All referenced studies used the MFQ-1 questionnaire, except for Atari et al. (2023), which used the updated MFQ-2.

The introduction of the updated MFQ-2 [7] provides an opportunity to reassess these relationships with a more refined instrument. This study, therefore, aimed to verify these findings with the new measure. Currently, only one study has investigated age and gender differences with MFQ-2 [7]. This study confirmed positive associations between age and all moral foundations except equality and higher scores for women in fairness (MFQ-2 equality) and purity (see Tables 1 and 2). Men scored modestly higher in loyalty, authority, and the newly added proportionality foundation. Notably, gender differences in care were minimal.

Drawing on MFQ-2 findings and the broader MFQ-1 literature, we hypothesized that our study would show age-related increases across all foundations (effect sizes ranging from  .05 to  .25), except possibly equality, consistent with the distribution of age effects reported in large-scale MFQ-1 studies [15]. Given that proportionality is part of the binding domain in MFQ-2, we expected its age association to be comparable in magnitude to the other binding foundations. Regarding gender, we predicted women would score higher on the individualizing foundations (care and equality) and purity, while men would show higher scores on the binding foundations (proportionality, loyalty, and authority), consistent with prior MFQ-1 results and with Atari, Haidt (7) who reported a modest male advantage in MFQ-2 proportionality. We further expected a male advantage in proportionality because it aligns more closely with equality- or merit-based allocation; across economic-game paradigms, men tend to endorse merit more and women equality more [20,21].

## Method

### Participants

The present research draws upon two samples collected by Zakharin and Bates [10] and Zakharin and Bates [22]. The samples were collected using Prolific Academic, a widely used online platform for scientific research. The first sample consisted of 1,659 UK participants (825 females, 827 males, 7 other) with a mean age of 41.16 years (SD = 13.97). The second sample consisted of 830 US participants (415 females, 415 males), with a mean age of 39.42 years (SD = 14.90).

The present study is a secondary analysis of previously collected data that are publicly available in de-identified form. The original data collection was approved by the Psychology Research Ethics Committee at the University of Edinburgh. Informed consent was obtained from all participants electronically prior to data collection. The current analyses used only anonymized, publicly shared data and therefore did not involve additional participant contact or collection of identifiable information.

### Materials

Moral foundations were assessed with the MFQ-2 [7]. This instrument consists of 36 items, six for each foundation, with responses on a 5-point Likert scale ranging from 1: Does not describe me at all to 5: Describes me extremely well. Participants rate how well each statement describes them or their opinions.

### Procedure

Participants from both samples completed MFQ-2 online via a Qualtrics survey. To ensure the privacy of participants, all collected data were de-identified using anonymous codes, and no personally identifying information was obtained. The authors did not have access to any information that could identify individual participants during or after data collection. Informed consent was obtained from all participants. Subjects then completed the items. A small monetary compensation was provided through the Prolific Academic platform. All statistical analyses were completed in R [23] and *umx* [24].

## Results

### Distributions of moral foundations’ scores

**Fig 1:**
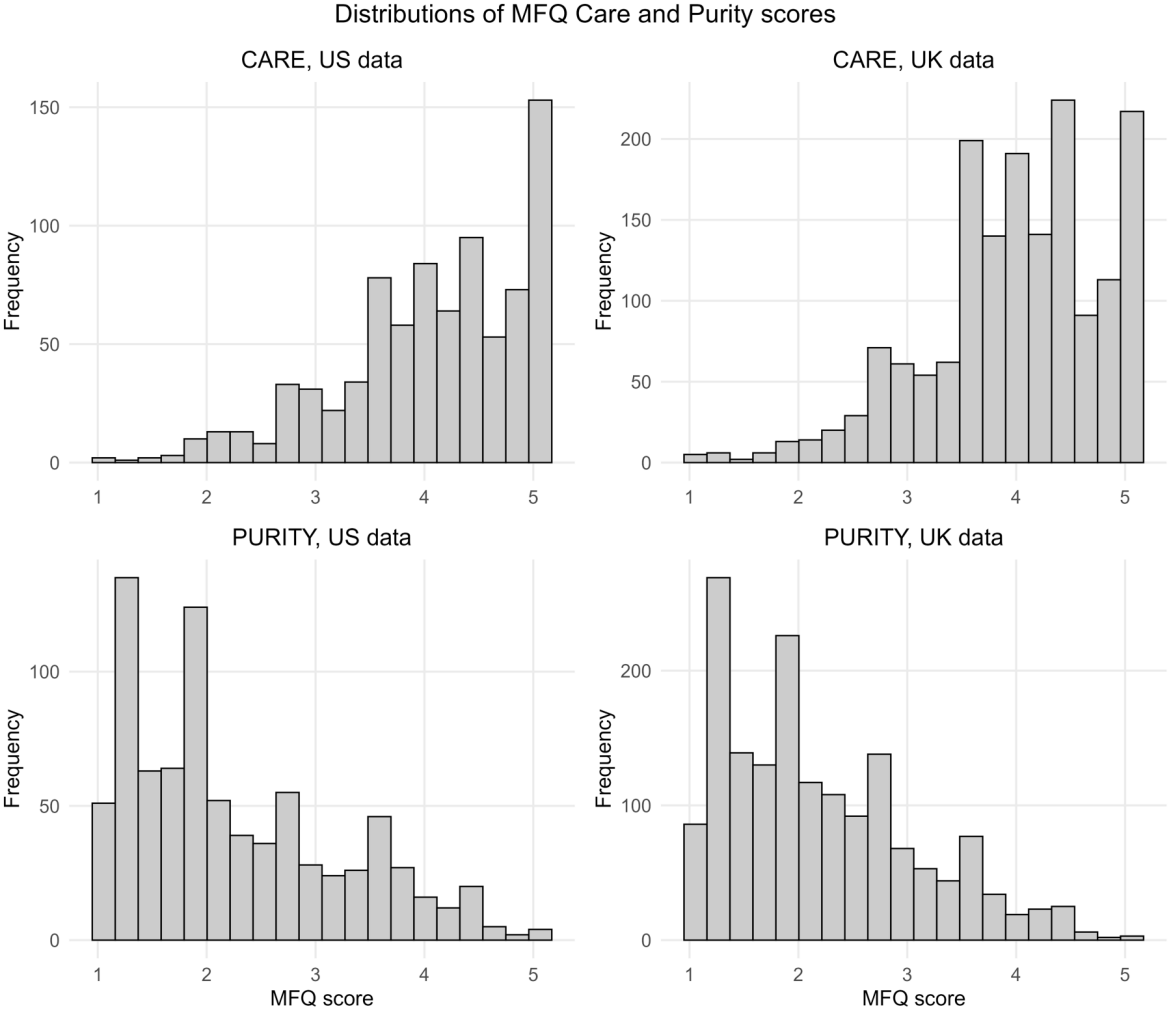
Distribution of scores for care and purity moral foundations across both datasets.

We first examined the distribution of scores in each MFQ-2 foundation. For each foundation (and the binding/individualizing composites), we summarized score distributions using the mean and SD and quantified distributional shape using skewness and kurtosis. We also inspected histograms to identify clustering near the minimum or maximum response options (floor/ceiling effects). As seen in Table 3 and graphically in Fig 1, care and purity demonstrated the most pronounced skewness, with care showing a strong right-skew while purity showed a strong left-skew. Histograms for all remaining foundations are presented in the supplementary materials. This observation suggests the presence of potential ceiling effects for care and floor effects for purity. For completeness, we also report kurtosis. All moral foundations except care showed a platykurtic distribution characterized by fewer scores falling close to the mean and thinner tails compared to a normal distribution.

**Table 3:** Descriptive statistics for each moral foundation and the broad binding and individualizing domains for US and UK samples separately*.*

Foundation	Mean		SD		Skew		Kurtosis	
	US	UK	US	UK	US	UK	US	UK
Care	4.05	3.98	0.83	0.78	−0.84	−0.80	0.26	0.58
Equality	2.88	2.94	1.09	0.97	0.09	0.03	−0.98	−0.77
Proportionality	3.63	3.63	0.78	0.74	−0.37	−0.36	−0.31	−0.19
Loyalty	2.81	2.88	0.99	0.93	0.34	0.12	−0.74	−0.71
Authority	3.01	3.16	1.02	0.95	0.15	−0.15	−0.9	−0.76
Purity	2.26	2.20	0.97	0.87	0.72	0.73	−0.42	−0.18
Binding	2.93	2.97	0.79	0.72	0.29	0.06	−0.66	−0.58
Individualizing	3.46	3.46	0.81	0.74	−0.19	−0.25	−0.53	−0.25

### Age and gender effects on moral foundations scores

**Fig 2:**
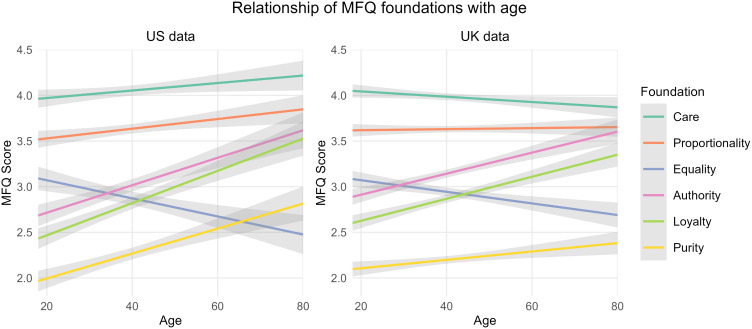
Relationship of foundation scores with age in the US and UK samples.

Next, we examined the effects of age, testing the hypothesis that scores on all moral foundations would increase with age, as reported by Castilla-Estévez and Blázquez-Rincón (15). In linear regression models with age predicting each moral foundation in turn (see Table 4), significant increases with age were found for the binding domain and for each of its four constituent foundations in both samples, except for proportionality, which showed the expected positive relationship with age in the US but not in the UK sample. Contrary to expectation, significant reductions in scores were obtained with age for individualizing and its constituent equality concern foundation. Scores on the care foundation showed no significant association with age. The trendlines for age and scores across all six moral foundations for the US and UK samples are shown in Fig 2.

**Table 4:** Standardized effects of age and sex on moral foundations scores in the US and UK samples.

Foundation	Age		Sex	
	US	UK	US	UK
**Individualizing**	−.08*	−.08***	−.19***	−.15***
Care	.05	−.04	−.17***	−.16***
Equality	−.15***	−.09***	−.16***	−.09***
**Binding**	.26***	.14***	.11***	.06*
Proportionality	.12***	.01	.14***	.10*
Loyalty	.28***	.18***	.13***	.07**
Authority	.23***	.17***	.13***	.06*
Purity	.21***	.07**	−.01	−.02

*Note*: For sex effects, positive *β* values reflect higher scores for males relative to females. Significance levels are indicated as follows: ***p < .001, **p < .01, *p < .05.

Regarding gender differences, as predicted, women scored significantly higher than men on individualizing foundations in both samples (see Table 4). The expected female superiority for purity, however, was not found. Instead, no significant gender differences were observed for purity in either sample. For loyalty and authority, the predicted higher scores for males were found in both samples. Based on the expectation for loyalty and authority, we had predicted that proportionality would also show a male advantage, which was supported (see Table 4). Given the number of comparisons across six moral foundations, two predictors (age, gender), and two samples, we prioritized the magnitude and consistency of standardized coefficients (β) over reliance on individual p-values. Nonetheless, we note that the majority of primary effects reported below survive a Bonferroni-corrected threshold of α = .05/12 = .004 per sample, and effects that replicated across both samples provide stronger evidence than any single test.

## Discussion

Our study revealed significant age and gender differences in moral foundations, along with notable response distributions for care and purity. Care showed a ceiling effect with modal responses clustered near the maximum possible score, while purity displayed a floor effect with modal responses near the minimum. Examining age effects, we found increases in all binding foundations and decreases in support for equality with age, while care scores remained stable across the age range. Regarding the effects of gender, as predicted, women scored higher on care and equality and lower on three out of four binding foundations (authority, loyalty, proportionality). Contrary to the prediction, no gender differences in purity were found.

Comparing our results to those based on the MFQ-1 [15], average effect sizes in our study were generally larger, perhaps suggesting that the improved measurement characteristics of the MFQ-2 enhanced its sensitivity. Specifically, correlations between age and binding foundations averaged approximately  .16 in our current MFQ-2 data, compared to  .06 to  .08 in the meta-analysis of MFQ-1 data. The directions of the effects, however, differed considerably in places from what had been observed for the MFQ-1.

The MFQ-1 and 2 differ most clearly in their treatment of fairness, with the MFQ-2 breaking the old fairness foundation into two independent dimensions of equality and proportionality. Whereas Castilla-Estévez and Blázquez-Rincón (15) found that scores on the old fairness domain increased with age, for the MFQ-2-based data in the present study, proportionality increased, but equality scores *decreased* with age, confirming Atari, Haidt (7) findings. This difference in sign of the age effects on equality and proportionality previously combined into a single foundation supports the more refined two-factor model of fairness provided by the MFQ-2. Turning to the second individualizing foundation, care, no significant effects of age were found, and the small differences observed oscillated around zero, with opposite sign in the two samples, suggesting that this foundation is unaffected by age.

While the effects of age on binding foundations may appear modest (r ~ .18), it is important to place them in the context of the long human lifespan where their practical significance becomes apparent. Each person transits through ages – at one time, we are infants, teens, and, hopefully, make our three score and ten or more. Across this wide range, even small annual changes lead to large cumulative gaps. For example, the expected difference in binding foundation scores between an 18-year-old and an 80-year-old was 0.78 SD in our sample. These differences, accumulated over decades, thus result in substantially different moral worldviews between younger and older adults.

These divergences have tangible societal implications. The sharp age-related decline in Equality concerns, combined with the rise in Binding foundations, suggests a mechanism for the well-documented conservative shift associated with aging. Furthermore, the 0.78 SD gap in binding scores between the youngest and oldest cohorts provides a quantitative basis for current intergenerational conflicts (’culture wars’). Where younger cohorts may view the prioritization of tradition and authority as inherently oppressive, older cohorts may view the dismissal of these foundations as a failure of moral duty. Such foundation-level disconnects complicate cross-generational political consensus. These findings are consistent with binding foundations reinforcing group loyalty, respect for authority, and traditional values. As individuals accumulate life experience, they may develop a greater appreciation for the role of authority, tradition, and established social hierarchies in maintaining a well-functioning society, leading to stronger endorsement of binding foundations.

Regarding gender disparities, the observed pattern aligns well with previous findings of higher individualizing and lower binding scores in women found in studies using the MFQ-1 [e.g., 18]. However, unlike previous studies that reported women scoring higher than men on purity, we found no evidence of gender differences here. This discrepancy may be explained by improvements in the measurement of purity foundation in MFQ-2, which excludes the disgust-related items present in MFQ-1. On this view, the earlier gender differences may have reflected women's higher disgust sensitivity, a well-known effect [25,26]. Thus, women tended to prioritize care, egalitarian outcomes and oppose meritocratic or proportional division, while men placed greater weight than women on loyalty, obedience to authority, and meritocratic forms of fairness. While modest in size, these gender differences, coupled with significant changes in gender roles in society, may have had a significant contribution to societal shifts toward more egalitarian and less hierarchical forms of governance.

These findings underscore the importance of considering both maturational and societal drivers. Maturational mechanisms likely include ‘social investment’ processes, where taking on adult roles (parenthood, employment) necessitates increased valuation of stability, authority, and loyalty. Simultaneously, societal drivers likely reflect the ‘Flynn effect for morality’ or broad secular liberalization, pushing cohort means toward higher Care and lower Purity. Given their shared cultural and economic structures, we expect these dual pressures to operate similarly across US and UK contexts, though their relative strength may vary with national factors such as religiosity and political culture.

Finally, in the results of distribution analyses, we found clear evidence of floor and ceiling effects. As shown in Fig 1, care and purity showed almost mirror-image shifts – with care having a modal response near the ceiling, while for purity, the modal response was almost the lowest possible score on the scale. Thus, in addition to age effects on binding foundations and reduced concerns for equality with cumulative effects nearing 1SD across the lifespan, we find evidence for population-level shifts of moral foundations scores downward for purity and upward for care.

### Limitations and future directions

Several limitations of this study suggest promising directions for future research. First, the associations we report are between-person age differences and cannot be taken as evidence of within-person developmental (maturational) change. Age is confounded with birth cohort and historical period: older participants differ not only in age but also in the social and cultural context in which they were raised. Thus, the observed age-related increase in binding foundations (and decrease in equality) could reflect maturation, cohort-based socialization, or both. Longitudinal—ideally cohort-sequential—data are needed to separate age, cohort, and period influences. Second, while self-report measures like the MFQ-2 have established utility, incorporating behavioral measures of moral and ethical decision-making would provide valuable complementary evidence.

Finally, our focus on US and UK samples limits generalizability, as cultures imbue moral values with different significance [7,13]. Future work should examine these patterns in diverse cultural contexts. Additionally, the observed floor and ceiling effects in purity and care scores may reflect broader societal influences that deserve systematic investigation, including socioeconomic factors, educational trends [27,28], and religious engagement. Historiographic approaches could help illuminate these sociocultural dynamics, for instance by analyzing moral themes in historical literature and other cultural artifacts [29].

## Conclusion

In conclusion, in two studies, we found increases in all binding foundations and decreases in support for equality with age, while care scores remained stable across the age range. For gender, women, as predicted, scored higher on both care and equality and lower on three out of four binding foundations (authority, loyalty, proportionality). Contrary to the prediction, no gender differences in purity were found. These findings underscore the importance of considering maturational [30,31] and societal [32] drivers of moral values.

## Supporting information

S1 File MFQ-1 items and domains. This file lists the items from the original Moral Foundations Questionnaire (MFQ-1), grouped by moral foundation/domain.
(DOCX)

S2 File MFQ-2 items and domains. This file lists the items from the Moral Foundations Questionnaire-2 (MFQ-2), grouped by moral foundation/domain.
(DOCX)
