# Supplementary material for: Age, gender, and score distributions of moral foundations
Source: PLoS One. 2026 Jul 1;21(7):e0352584. doi: 10.1371/journal.pone.0352584 (PMC13322558; doi:10.1371/journal.pone.0352584)
Supplement: S1 File — This file lists the items from the original Moral Foundations Questionnaire (MFQ-1), grouped by moral foundation/domain. (DOCX) [file pone.0352584.s001.docx]

**MORAL FOUNDATIONS QUESTIONNAIRE-1**

**(MFQ-1)**

**Reference:** Graham, J., Nosek, B. A., Haidt, J., Iyer, R., Koleva, S., & Ditto, P. H. (2011). Mapping the moral domain. *Journal of Personality and Social Psychology* 101(2): 366-385. https://doi.org/10.1037/a0021847

MFQ-1 contains two parts. In each moral foundation, Items 1-3 belong to the Relevance scale, and Items 4-6 belong to the Judgment scale.

**Relevance Scale**

When you decide whether something is right or wrong, to what extent are the following considerations relevant to your thinking? Please rate each statement using this scale:

**Response Options:**

o *Not at all relevant*

o *Not very relevant*

o *Slightly relevant*

o *Somewhat relevant*

o *Very relevant*

o *Extremely relevant*

**Judgment Scale**

Please read the following sentences and indicate your agreement or disagreement using the following response options:

**Response Options:**

o *Strongly disagree*

o *Moderately disagree*

o *Slightly disagree*

o *Slightly agree*

o *Moderately agree*

o *Strongly agree*

**Harm/Care**

Items 1-3 are Relevance; Items 4-6 are Judgment.

1. Whether or not someone suffered emotionally

2. Whether or not someone cared for someone weak or vulnerable

3. Whether or not someone was cruel

4. Compassion for those who are suffering is the most crucial virtue

5. One of the worst things a person could do is hurt a defenseless animal

6. It can never be right to kill a human being

**Fairness**

Items 1-3 are Relevance; Items 4-6 are Judgment.

1. Whether or not some people were treated differently than others

2. Whether or not someone acted unfairly

3. Whether or not someone was denied his or her rights

4. When the government makes laws, the number one principle should be ensuring that everyone is treated fairly

5. Justice is the most important requirement for a society

6. I think it’s morally wrong that rich children inherit a lot of money while poor children inherit nothing

**Loyalty**

Items 1-3 are Relevance; Items 4-6 are Judgment.

1. Whether or not someone’s action showed love for his or her country

2. Whether or not someone did something to betray his or her group

3. Whether or not someone showed a lack of loyalty

4. I am proud of my country’s history

5. People should be loyal to their family members, even when they have done something wrong

6. It is more important to be a team player than to express oneself

**Authority**

Items 1-3 are Relevance; Items 4-6 are Judgment.

1. Whether or not someone showed a lack of respect for authority

2. Whether or not someone conformed to the traditions of society

3. Whether or not an action caused chaos or disorder

4. Respect for authority is something all children need to learn

5. Men and women each have different roles to play in society

6. If I were a soldier and disagreed with my commanding officer’s orders, I would obey anyway because that is my duty

**Purity**

Items 1-3 are Relevance; Items 4-6 are Judgment.

1. Whether or not someone violated standards of purity and decency

2. Whether or not someone did something disgusting

3. Whether or not someone acted in a way that God would approve of

4. People should not do things that are disgusting, even if no one is harmed

5. I would call some acts wrong on the grounds that they are unnatural

6. Chastity is an important and valuable virtue

**Filler Items (attention checks)**

Item 1 is Relevance; Item 2 is Judgment.

1. Whether or not someone was good at math

2. It is better to do good than to do bad
