# Supplementary material for: Age, gender, and score distributions of moral foundations
Source: PLoS One. 2026 Jul 1;21(7):e0352584. doi: 10.1371/journal.pone.0352584 (PMC13322558; doi:10.1371/journal.pone.0352584)
Supplement: S2 File — This file lists the items from the Moral Foundations Questionnaire-2 (MFQ-2), grouped by moral foundation/domain. (DOCX) [file pone.0352584.s002.docx]

**MORAL FOUNDATIONS QUESTIONNAIRE-2**

**(MFQ-2)**

Reference: Atari, M., et al. (2023). Morality beyond the WEIRD: How the nomological network of morality varies across cultures. *Journal of Personality and Social Psychology* **125**: 1157-1188.

For each of the statements below, please indicate how well each statement describes you or your opinions.

**Response Options:**

- *Does not describe me at all*
- *Slightly describes me*
- *Moderately describes me*
- *Describes me fairly well*
- *Describes me extremely well*

**Care**

1. Caring for people who have suffered is an important virtue.
2. I believe that compassion for those who are suffering is one of the most crucial virtues.
3. We should all care for people who are in emotional pain.
4. I am empathetic toward those people who have suffered in their lives.
5. Everyone should try to comfort people who are going through something hard.
6. It pains me when I see someone ignoring the needs of another human being.

**Equality**

1. The world would be a better place if everyone made the same amount of money.
2. Our society would have fewer problems if people had the same income.
3. I believe that everyone should be given the same quantity of resources in life.
4. I believe it would be ideal if everyone in society wound up with roughly the same amount of money.
5. When people work together toward a common goal, they should share the rewards equally, even if some worked harder on it.
6. I get upset when some people have a lot more money than others in my country.

**Proportionality**

1. I think people who are more hard-working should end up with more money.
2. I think people should be rewarded in proportion to what they contribute.
3. The effort a worker puts into a job ought to be reflected in the size of a raise they receive.
4. It makes me happy when people are recognized on their merits.
5. In a fair society, those who work hard should live with higher standards of living.
6. I feel good when I see cheaters get caught and punished.

**Loyalty**

1. I think children should be taught to be loyal to their country.
2. It upsets me when people have no loyalty to their country.
3. Everyone should love their own community.
4. Everyone should defend their country, if called upon.
5. Everyone should feel proud when a person in their community wins in an international competition.
6. I believe the strength of a sports team comes from the loyalty of its members to each other.

**Authority**

1. I think it is important for societies to cherish their traditional values.
2. I feel that most traditions serve a valuable function in keeping society orderly
3. I think obedience to parents is an important virtue.
4. We all need to learn from our elders.
5. I believe that one of the most important values to teach children is to have respect for authority.
6. I think having a strong leader is good for society.

**Purity**

1. I think the human body should be treated like a temple, housing something sacred within.
2. I believe chastity is an important virtue.
3. It upsets me when people use foul language like it is nothing.
4. If I found out that an acquaintance had an unusual but harmless sexual fetish I would feel uneasy about them.
5. People should try to use natural medicines rather than chemically identical human-made ones.
6. I admire people who keep their virginity until marriage.
