# Supplementary material for: Age, gender, and score distributions of moral foundations
Source: PLoS One. 2026 Jul 1;21(7):e0352584. doi: 10.1371/journal.pone.0352584 (PMC13322558; doi:10.1371/journal.pone.0352584)
Supplement: Attachment — Submitted filename: PONE-D-24-60347_R1.pdf [file pone.0352584.s004.pdf]

PLOS One

Age, gender, and score distributions of moral foundations

--Manuscript Draft--

**Manuscript Number:**	PONE-D-24-60347R1
**Article Type:**	Research Article
**Full Title:**	Age, gender, and score distributions of moral foundations
**Short Title:**	Age, gender, and moral foundations
**Corresponding Author:**	Michael Zakharin, Ph.D.<br>The University of Edinburgh<br>Edinburgh, UNITED KINGDOM OF GREAT BRITAIN AND NORTHERN IRELAND
**Keywords:**	moral foundations; age; Gender; Generations; Equality; Purity; Proportionality; authority; loyalty; care
**Abstract:**	Generational differences in moral foundations may provide crucial insights into societal change. Using the recently developed Moral Foundations Questionnaire (MFQ-2), we examined age and gender effects on moral values in US (N = 835) and UK (N = 1,659) samples. We found robust age-related increases in binding foundations (purity, loyalty, authority, and proportionality). While these changes were modest annually, they accumulated to substantial differences (~0.75 SD) between the youngest and oldest participants. Among individualizing foundations, the preference for equality decreased with age, while the harm foundation remained stable. Gender differences also emerged, with women scoring higher than men on individualizing foundations while men scored higher on binding foundations. Significant skew was observed in two foundations: care scores approached ceiling levels, while purity scores clustered near floor levels. The implications of these demographic differences in interaction with social changes are discussed.
**Order of Authors:**	Michael Zakharin, PhD
	Timothy C. Bates, Professor
**Response to Reviewers:**	Dear Dr. Carrasco-Farré,<br>Thank you for the opportunity to revise our manuscript. We are grateful to you and the reviewers for the positive evaluation of our design and methodology, and for the constructive feedback regarding the theoretical framing and discussion of implications. We have addressed all points raised. Specifically, we have:<br>1.Clarified the theoretical basis for our hypotheses regarding proportionality and effect sizes.<br>2.Expanded the discussion on the societal and political implications of these age/gender gaps.<br>3.Elaborated on the "maturational vs. societal" mechanisms in the Conclusion.<br>4.Provided detailed definitions of the six MFQ-2 domains and clarified the "distributional effects" terminology throughout the manuscript.<br>5.Added explicit text regarding cross-sectional design limitations.<br>Below is a detailed point-by-point response to the Academic Editor and Reviewers.<br>____<br>Response to the Academic Editor<br>Editor Point 1: Clarify and motivate the hypotheses more explicitly.<br>"As Reviewer 1 notes, it would be helpful to expand on the reasoning behind specific predictions, such as why proportionality was expected to show a male advantage, and why you anticipated age-related increases across all foundations with effect sizes in the .05–.25 range."<br>Response:<br>We agree that the specific predictions for Proportionality (a new foundation in MFQ-2) and the specific effect size estimates required stronger grounding in the introduction.<br>•Regarding Proportionality: We have added text explaining that proportionality aligns with "equity" or meritocratic allocation, a domain where men consistently score higher in evolutionary psychology and economic game literature, contrasting with the "equality" domain where women typically score higher.<br>•Regarding Effect Sizes: We have clarified that the range estimate (.05–.25) was

Powered by Editorial Manager® and ProduXion Manager® from Aries Systems Corporation

derived directly from the meta-analytic baselines established by Castilla-Estévez & Blázquez-Rincón (2021) and the specific findings of Milojev et al. (2014).
Change to Manuscript:
Original text: " Drawing on these MFQ-2 findings and the broader MFQ-1 literature, we hypothesized..."
New text: "Drawing on MFQ-2 findings and the broader MFQ-1 literature, we hypothesized that our study would show age-related increases across all foundations (effect sizes ranging from .05 to .25), except possibly equality, consistent with the distribution of age effects reported in large-scale MFQ-1 studies (Castilla-Estévez & Blázquez-Rincón, 2021). Given that proportionality is part of the binding domain in MFQ-2, we expected its age association to be comparable in magnitude to the other binding foundations. Regarding gender, we predicted women would score higher on the individualizing foundations (care and equality) and purity, while men would show higher scores on the binding foundations (proportionality, loyalty, and authority), consistent with prior MFQ-1 results and with Atari et al. (2023) who reported a modest male advantage in MFQ-2 proportionality. We further expected a male advantage in proportionality because it aligns more closely with equality- or merit-based allocation; across economic-game paradigms, men tend to endorse merit more and women equality more (Capraro, 2020; Fehr et al., 2006)." (page 6, lines 119 - 130)
Editor Point 2: Elaborate the implications of the observed age and gender differences.
"Reviewer 1 also asks for more discussion of how these differences in moral foundations may translate into broader social and political outcomes... Deepening this part of the Discussion will help readers see the societal relevance of your findings."
Response:
We have expanded the Discussion to explicitly link these psychometric differences to real-world phenomena, specifically political polarization and intergenerational value conflict (e.g., "culture wars").
Change to Manuscript:
New text: "These divergences have tangible societal implications. The sharp age-related decline in Equality concerns, combined with the rise in Binding foundations, suggests a mechanism for the well-documented conservative shift associated with aging. Furthermore, the 0.78 SD gap in binding scores between the youngest and oldest cohorts provides a quantitative basis for current intergenerational conflicts ('culture wars'). Where younger cohorts may view the prioritization of tradition and authority as inherently oppressive, older cohorts may view the dismissal of these foundations as a failure of moral duty. Such foundation-level disconnects complicate cross-generational political consensus." (page 12, lines 251 - 258)
Editor Point 3: Develop the point about maturational versus societal causes.
"Please expand this argument: outline what kinds of maturational mechanisms you have in mind, what societal changes might be relevant, and whether you would expect these to operate similarly or differently in the US and UK contexts."
Response:
We have expanded the Conclusion to distinguish between Social Investment Theory (maturation) and secular liberalization (societal change). We also noted that given the shared "WEIRD" (Western, Educated, Industrialized, Rich, Democratic) status of the US and UK, we expect similar mechanisms in both, though the baseline levels might differ.
Change to Manuscript (Conclusion):
Original text: "Findings underscore the importance of considering both maturational... and societal... causes as drivers of changes in moral values."
New text: "These findings underscore the importance of considering both maturational and societal drivers. Maturational mechanisms likely include 'social investment' processes, where taking on adult roles (parenthood, employment) necessitates increased valuation of stability, authority, and loyalty. Simultaneously, societal drivers likely reflect the 'Flynn effect for morality' or broad secular liberalization, pushing cohort means toward higher Care and lower Purity. Given their shared cultural and economic structures, we expect these dual pressures to operate similarly across US and UK contexts, though their relative strength may vary with national factors such as religiosity and political culture." (page 13, lines 275 - 282)
Editor Point 4: Provide more background on the MFQ-1/MFQ-2 and the moral domains.
"Please elaborate briefly on the six moral domains... and clarify how MFQ-2 differs from MFQ-1... provide the MFQ-1 and MFQ-2 item sets as Supporting Information files..."
Response:

Powered by Editorial Manager® and ProduXion Manager® from Aries Systems Corporation

	We have expanded the introduction to clearly define the six domains. We have also uploaded the full item sets for MFQ-1 and MFQ-2 as a Supporting Information file (S1_File_MFQ_Items.pdf and S2_File_MFQ -2_Items.pdf) as requested.<br>Change to Manuscript:<br>Original text: "The MFQ-2 differs from the MFQ-1 in two notable ways…"<br>New text: " Moral foundations theory (MFT; Atari et al., 2023; Graham et al., 2011; Haidt & Joseph, 2004) proposes that moral judgment is supported by multiple partially distinct concerns rather than a single moral principle. In this paper we focus on the six foundations assessed by the MFQ-2: care, equality, proportionality, loyalty, authority, and purity. Care captures concern for others' suffering and harm prevention. Equality reflects egalitarian concern that people should be treated similarly and have similar outcomes. Proportionality reflects merit-based fairness - benefits and punishments should track effort, contribution, or responsibility (conceptually related to mutualism; Baumard et al., 2013; Lin & Bates, 2022). Loyalty concerns commitment to one's group and aversion to betrayal. Authority concerns respect for legitimate hierarchy, tradition, and role-based duties. Purity concerns contamination and sanctity, including bodily and spiritual cleanliness and self-control. These foundations are often summarized into higher-order clusters in Western samples, with individualizing concerns (care, equality) emphasizing protection and equal regard for individuals, and binding concerns (loyalty, authority, purity, and proportionality) emphasizing group cohesion, order, and norm enforcement. (Atari et al., 2023; Graham et al., 2011).<br>Moral foundations are commonly measured with the MFQ-1 (Graham et al., 2011) and the updated MFQ-2 (Atari et al., 2023). The key conceptual difference is that MFQ-1 assesses five foundations, including a single fairness scale, whereas recently developed MFQ-2 assesses six foundations by splitting fairness into two separable constructs: equality and proportionality. In addition, MFQ-2 replaces the original MFQ-1 items with newly written or rephrased items, intended to improve clarity and content coverage and to better recover the expected factor structure in contemporary samples (Atari et al., 2023; Zakharin & Bates, 2023), addressing well-documented psychometric limitations of MFQ-1 in some datasets (e.g. Davies et al., 2014; Doğruyol et al., 2019; Iurino & Saucier, 2018; Kivikangas et al., 2017).The complete item sets for MFQ-1 and MFQ-2 are provided in the Supporting Information." (page 4, lines 67 - 74)<br>Editor Point 5: Clarify the "distributional effects" framing.<br>"The title and framing emphasise "distributional effects," but the meaning of this term... could be described more explicitly."<br>Response:<br>We used "distributional effects" to refer specifically to the shape of the score distributions (skewness/kurtosis) and the identification of ceiling/floor effects, rather than variance heterogeneity. We have clarified this definition in the Introduction and Results to avoid ambiguity. We also changed the title to "Age, gender, and score distributions of moral foundations"<br>Change to Manuscript:<br>New text (Introduction): "... to describe mean differences in moral foundations by age and gender and the shape of MFQ-2 scores in the population—specifically examining distributional properties such as skew and ceiling/floor effects—and the changes exhibited with age." (page 3, lines 49 - 52)<br>New text (Results): "For each foundation (and the binding/individualizing composites), we summarized score distributions using the mean and SD and quantified distributional shape using skewness and kurtosis. We also inspected histograms to identify clustering near the minimum or maximum response options (floor/ceiling effects)" (page 9, lines 167 - 171)<br>Editor Point 6: Multiple comparisons and effect size interpretation.<br>"State how you addressed the issue of multiple testing... consider adding a short paragraph in the Discussion that contextualises the magnitude of the observed effects"<br>Response:<br>Given the large sample size (N > 2400 total) and the exploratory nature of the demographic mapping, we prioritized estimation of effect sizes (beta) over strict p-value thresholding, though we note that our key findings are significant at $p < .001$, robust to Bonferroni correction for the 12 primary tests (6 domains × 2 demographic variables).<br>Change to Manuscript (Results):<br>New text: "Given the number of comparisons across six moral foundations, two predictors (age, gender), and two samples, we prioritized the magnitude and consistency of standardized coefficients (β) over reliance on individual p-values.

Powered by Editorial Manager® and ProduXion Manager® from Aries Systems Corporation

	Nonetheless, we note that the majority of primary effects reported below survive a Bonferroni-corrected threshold of $\alpha = .05/12 = .004$ per sample, and effects that replicated across both samples provide stronger evidence than any single test." (page 10, lines 204 - 209)<br>Editor Point 7: Age, cohort, and design limitations.<br>"Because the data are cross-sectional, age effects are potentially confounded with cohort and period effects... acknowledge this explicitly in the Limitations section"<br>Response:<br>We have reinforced the Limitations section to explicitly state the Age/Period/Cohort confound.<br>Change to Manuscript:<br>Original text: "Our cross-sectional design cannot disentangle age effects from cohort effects..."<br>New text: "The associations we report are between-person age differences and cannot be taken as evidence of within-person developmental (maturational) change. Age is confounded with birth cohort and historical period: older participants differ not only in age but also in the social and cultural context in which they were raised. Thus, the observed age-related increase in binding foundations (and decrease in equality) could reflect maturation, cohort-based socialization, or both. Longitudinal—ideally cohort-sequential—data are needed to separate age, cohort, and period influences." (page 13, lines 291 - 297)<br>______________________________________<br>Response to Reviewers<br>Reviewer #1<br>We thank Reviewer 1 for their assessment that the manuscript is "well-written and well-designed"15.<br>R1 Point 1: Explain hypotheses (Proportionality male advantage, effect sizes). 16<br>Response: As detailed in our response to the Editor (Point 1), we have revised the Introduction to explicitly ground the Proportionality hypothesis in literature suggesting male preferences for equity/meritocracy over equality and justified the effect size prediction based on the Castilla-Estévez meta-analysis.<br>R1 Point 2: Discuss repercussions/implications.<br>Response: We have expanded the Discussion to address political affiliations and intergenerational value conflict, as requested. (See response to Editor Point 2).<br>R1 Point 3: Expand on maturational vs. societal causes.<br>Response: We have elaborated on the Conclusion to contrast social investment mechanisms against secular shifts in values. (See response to Editor Point 3).<br>Reviewer #2<br>We thank Reviewer 2 for their comments regarding the solid analysis.<br>R2 Point 1: Provide a copy of the MFQ-1 and MFQ-2. 20<br>Response: We have created a Supporting Information files containing the items for the MFQ-1 and MFQ-2 to assist readers in understanding the instrument.<br>R2 Point 2: Elaborate on the different domains.<br>Response: We have revised the Introduction to provide distinct, conceptual definitions for Care, Equality, Proportionality, Loyalty, Authority, and Purity. (See response to Editor Point 4).<br>______________________________________<br>Specific Ethics Statement Update<br>Per Journal Requirement #222, we have updated the Methods section:<br>" The present study is a secondary analysis of previously collected data that are publicly available in de-identified form. The original data collection was approved by the Psychology Research Ethics Committee at the University of Edinburgh. Informed consent was obtained from all participants electronically prior to data collection. The current analyses used only anonymized, publicly shared data and therefore did not involve additional participant contact or collection of identifiable information."<br>Data Availability<br>Data remains available as per the original submission.<br>We believe these revisions significantly strengthen the manuscript and look forward to your decision.<br>Sincerely,<br>Michael Zakharin & Timothy C. Bates<br>Department of Psychology, University of Edinburgh
**Additional Information:**	

Powered by Editorial Manager® and ProduXion Manager® from Aries Systems Corporation

Question	Response
**Financial Disclosure**<br><br>Enter a financial disclosure statement that describes the sources of funding for the work included in this submission. Review the submission guidelines for detailed requirements. View published research articles from *PLOS ONE* for specific examples.<br><br>This statement is required for submission and **will appear in the published article** if the submission is accepted. Please make sure it is accurate.<br><br>**Funded studies**<br>Enter a statement with the following details:<br>• Initials of the authors who received each award<br>• Grant numbers awarded to each author<br>• The full name of each funder<br>• URL of each funder website<br>• Did the sponsors or funders play any role in the study design, data collection and analysis, decision to publish, or preparation of the manuscript?<br><br>Did you receive funding for this work?	The author(s) received no specific funding for this work.
**Competing Interests**<br><br>Use the instructions below to enter a competing interest statement for this submission. On behalf of all authors, disclose any competing interests that could be perceived to bias this work—acknowledging all financial support and any other relevant financial or non-financial competing interests.<br><br>This statement is required for submission and **will appear in the published article** if the submission is accepted. Please make sure it is accurate and that any funding sources listed in your Funding Information later in the submission form are also	The authors have declared that no competing interests exist.

declared in your Financial Disclosure statement.<br><br>View published research articles from [PLOS ONE](#) for specific examples.<br><br>**NO authors have competing interests**<br><br>Enter: *The authors have declared that no competing interests exist.*<br><br>**Authors with competing interests**<br><br>Enter competing interest details beginning with this statement:<br><br>*I have read the journal's policy and the authors of this manuscript have the following competing interests: [insert competing interests here]*<br><br>* typeset	
**Ethics Statement**<br><br>Enter an ethics statement for this submission. This statement is required if the study involved:<br><br>• Human participants<br>• Human specimens or tissue<br>• Vertebrate animals or cephalopods<br>• Vertebrate embryos or tissues<br>• Field research<br><br>Write "N/A" if the submission does not require an ethics statement.<br><br>General guidance is provided below. Consult the submission guidelines for detailed instructions. **Make sure that all information entered here is included in the Methods section of the manuscript.**	The study was approved by the Psychology Research Ethics Committee at the School of Philosophy, Psychology & Language Sciences at the University of Edinburgh, Ref No: 413-2122/2. A written consent (using online form) was obtained from participants.

Powered by Editorial Manager® and ProduXion Manager® from Aries Systems Corporation

**Format for specific study types**<br><br>**Human Subject Research (involving human participants and/or tissue)**<br>• Give the name of the institutional review board or ethics committee that approved the study<br>• Include the approval number and/or a statement indicating approval of this research<br>• Indicate the form of consent obtained (written/oral) or the reason that consent was not obtained (e.g. the data were analyzed anonymously)<br><br>**Animal Research (involving vertebrate animals, embryos or tissues)**<br>• Provide the name of the Institutional Animal Care and Use Committee (IACUC) or other relevant ethics board that reviewed the study protocol, and indicate whether they approved this research or granted a formal waiver of ethical approval<br>• Include an approval number if one was obtained<br>• If the study involved *non-human primates*, add *additional details* about animal welfare and steps taken to ameliorate suffering<br>• If anesthesia, euthanasia, or any kind of animal sacrifice is part of the study, include briefly which substances and/or methods were applied<br><br>**Field Research**<br><br>Include the following details if this study involves the collection of plant, animal, or other materials from a natural setting:<br>• Field permit number<br>• Name of the institution or relevant body that granted permission	
**Data Availability**<br><br>Authors are required to make all data underlying the findings described fully available, without restriction, and from the time of publication. PLOS allows rare exceptions to address legal and ethical concerns. See the PLOS Data Policy and FAQ for detailed information.	Yes - all data are fully available without restriction

A Data Availability Statement describing where the data can be found is required at submission. Your answers to this question constitute the Data Availability Statement and **will be published in the article**, if accepted.<br><br>**Important:** Stating 'data available on request from the author' is not sufficient. If your data are only available upon request, select 'No' for the first question and explain your exceptional situation in the text box.<br><br>Do the authors confirm that all data underlying the findings described in their manuscript are fully available without restriction?	
**Describe where the data may be found in full sentences. If you are copying our sample text, replace any instances of XXX with the appropriate details.**<br><br>• If the data are **held or will be held in a public repository**, include URLs, accession numbers or DOIs. If this information will only be available after acceptance, indicate this by ticking the box below. For example: *All XXX files are available from the XXX database (accession number(s) XXX, XXX.)*.<br>• If the data are all contained **within the manuscript and/or Supporting Information files**, enter the following: *All relevant data are within the manuscript and its Supporting Information files.*<br>• If neither of these applies but you are able to provide **details of access elsewhere**, with or without limitations, please do so. For example:<br><br>*Data cannot be shared publicly because of [XXX]. Data are available from the XXX Institutional Data Access / Ethics Committee (contact via XXX) for researchers who meet the criteria for access to confidential data.*<br><br>*The data underlying the results presented in the study are available from (include the name of the third party*	The data and r code are publicly available at OSF site: https://osf.io/yk3qc/<br><br>DOI 10.17605/OSF.IO/YK3QC

and contact information or URL).<br>• This text is appropriate if the data are owned by a third party and authors do not have permission to share the data.<br><br>* typeset	
Additional data availability information:	

Powered by Editorial Manager® and ProduXion Manager® from Aries Systems Corporation

Manuscript

Click here to access/download;Manuscript;1 draft mfq age no author_clean.docx

Age, gender, and score distributions of moral foundations

Authors

Michael Zakharin and Timothy C Bates

Address

Department of Psychology, University of Edinburgh

7 George Square

Edinburgh EH8 9JZ

Telephone +44 131 650 1945

tim.bates@ed.ac.uk (Orcid: 0000-0002-1153-9007) or

michael.zakharin@vcuhealth.org (Orcid: 0000-0003-3689-4635)

Abstract

Claims of generational differences in moral values have important implications for understanding societal change. Using the recently developed Moral Foundations Questionnaire (MFQ-2), we examined age and gender effects on moral foundations in large US (N = 835) and UK (N = 1,659) samples. Across both nations, binding foundations (proportionality, loyalty, authority and purity) showed consistent age-related increases. While these yearly increments were modest, they accumulated to substantial differences between youngest and oldest participants, with binding scores approximately 0.75 standard deviations higher in older adults. Among individualizing foundations, the preference for equality decreased with age, while harm/care remained stable. Gender differences emerged systematically: women scored higher on individualizing foundations, while men showed elevated scores on binding foundations. We also observed significant distributional effects, with care scores clustering near the scale maximum and purity scores near the minimum. These demographic patterns suggest important dynamics in how moral values may shift across generations and between genders, with implications for understanding social change.

Introduction

“*Children began to be the tyrants, not the slaves, of their households. They no longer rose from their seats when an elder entered the room; they contradicted their parents, chattered before company, gobbled up the dainties at table, and committed various offences against Hellenic tastes, such as crossing their legs. They tyrannised over the paidagogoi and schoolmasters*” [1].

As noted by Freeman (1), ancient writers, particularly those from ancient Greece such as Aristophanes and Xenophon, argued that the young of their day lacked the moral rectitude they themselves had shown at their age. These persistent claims of generational moral differences warrant rigorous investigation, as documenting either the presence or absence of age-related variations in moral foundations carries significant implications for understanding societal changes. Differences between young and old, of course, may refer to very different phenomena and causes [2]. One possibility is that moral foundations exhibit reliable developmental change, as suggested by classic theories of moral development, which propose a stage-like process of increasing moral concern [3, 4]. Alternatively, change may affect the culture as a whole, impacting persons of all ages and manifesting as a shift in the mean score for the population. In such mean-shift cases, older generations who lived through an earlier, perhaps stricter (or more lax) time will observe that young people differ not only from their own youth but potentially from how their parents were at the same age. Here, we use the Moral Foundations Questionnaire- 2 (MFQ-2) to examine differences in the moral foundations in two new samples, one US and one UK, to describe mean differences in moral foundations by age and gender and the shape of MFQ-2 scores in the population—specifically examining distributional properties such as skew and ceiling/floor effects—and the changes exhibited with age. We first provide background on moral foundation theory and work on age and gender differences in foundation scores.

Moral foundations theory [MFT; 5, 6, 7] proposes that moral judgment is supported by multiple partially distinct concerns rather than a single moral principle. In this paper we focus on the six foundations assessed by the MFQ-2: care, equality, proportionality, loyalty, authority, and purity. Care captures concern for others’ suffering and harm prevention. Equality reflects egalitarian concern that people should be treated similarly and have similar outcomes. Proportionality reflects merit-based fairness - benefits and punishments should track effort, contribution, or responsibility, conceptually related to mutualism [8, 9]. Loyalty concerns commitment to one’s group and aversion to betrayal. Authority concerns respect for legitimate hierarchy, tradition, and role-based duties. Purity concerns contamination and sanctity, including bodily and spiritual cleanliness and self-control. These foundations are often summarized into

higher-order clusters in Western samples, with individualizing concerns (care, equality) emphasizing protection and equal regard for individuals, and binding concerns (loyalty, authority, purity, and proportionality) emphasizing group cohesion, order, and norm enforcement [6, 7].

Moral foundations are commonly measured with the MFQ-1 [6] and the updated MFQ-2 [7]. The key conceptual difference is that MFQ-1 assesses five foundations, including a single fairness scale, whereas recently developed MFQ-2 assesses six foundations by splitting fairness into two separable constructs: equality and proportionality. In addition, MFQ-2 replaces the original MFQ-1 items with newly written or rephrased items, intended to improve clarity and content coverage and to better recover the expected factor structure in contemporary samples [7, 10], addressing well-documented psychometric limitations of MFQ-1 in some datasets [e.g. 11, 12-14]. The complete item sets for MFQ-1 and MFQ-2 are provided in the Supporting Information.

Having described the MFT foundations and measures, we next turn to existing research on the relationships of age and sex to moral foundations, focussing on studies with large sample sizes that offer the most relevant and robust findings.

Age differences in moral foundations scores

The most comprehensive examination of age effects on moral foundations scores is the meta-analysis of 239 independent samples and 492,298 subjects using the MFQ-1, as reported by Castilla-Estévez and Blázquez-Rincón (15). Their analysis revealed a consistent albeit small (mean r= 0.06) age-related increase in moral concern across all foundations (see Table 1). Two studies not included in [15] support this finding, with Nilsson (16) replicating the increase in all foundations in a Swedish sample (N = 2,282), with around twice the effect size as reported by Castilla-Estévez and Blázquez-Rincón (15). In a New Zealand sample, Milojev, Osborne (17) also found that scores on all foundations increased with age, with a mean age effect of .21 and larger effects in binding than individualizing. These studies and their estimates of age effects on moral foundations are summarized in Table 1. While these effects may appear modest, it is important to note that small increases, when accumulated over many decades of life, result in substantial differences in scores between the youngest and oldest members of the population.

Table 1. Correlations between moral foundations and age across the previous studies.

	Individualizing		Binding			
Study	Care	Fairness/ Equality	Proportionality	Loyalty	Authority	Purity
Castilla-Estévez & Blázquez-Rincón (2021) N = 492,298	.08	.06	--	.04	.06	.07
Nilsson (2023) N = 2,282	.13	.07	--	.18	.07	.13
Milojev et al. (2014) N = 3635	.05	.15	--	.26	.29	.29
Atari et al. (2023; MFQ-2) N = 1410	.09	-.08	.04	.11	.12	.09

Gender differences in moral foundations scores

The most extensive study of gender differences in MFQ-1 scores comes from Study 1 of Atari, Lai and Dehghani (18), reporting data from the online international dataset hosted on YourMorals.org (N = 348,660, with 67 countries represented with the majority (N = 262,629) coming from the US). Atari, Lai and Dehghani (18) reported higher scores in women for care, fairness, and purity, with care showing the greatest disparity. However, no significant differences were observed for loyalty or authority. These patterns were confirmed in an independent international sample (N = 11,969), although the gender gap in care scores was narrower, more closely matching the differences observed in fairness and purity (see Table 2). Similar results were also reported by Van Leeuwen, Dukes (19) using an international sample primarily from Western countries (N = 2,478), see Table 2. Again, women scored higher on care, fairness, and purity, and men scored higher on loyalty, with no difference found in authority. Likewise, in a Swedish sample (N = 2,282), Nilsson (16) replicated gender differences in care and fairness alongside a slight male advantage in authority, though the gender effect on purity did not hold. Finally, a study from New Zealand (N = 3,635) by Milojev, Osborne (17) echoed the gender discrepancies in care and fairness and found modest but significant gender differences in the binding foundations favouring men, with no significant effects found for purity.

The introduction of the updated MFQ-2 [7] provides an opportunity to reassess these relationships with a more refined instrument. This study, therefore, aimed to verify these findings with the new measure. Currently, only one study has investigated age and gender differences with MFQ-2 [7]. This study confirmed positive associations between age and all moral foundations except equality and higher scores for women in fairness (MFQ-2 equality) and purity (see Tables 1 and 2). Men scored modestly higher in loyalty, authority, and the newly added proportionality foundation. Notably, gender differences in care were minimal.

Drawing on MFQ-2 findings and the broader MFQ-1 literature, we hypothesized that our study would show age-related increases across all foundations (effect sizes ranging from .05 to .25), except possibly equality, consistent with the distribution of age effects reported in large-scale MFQ-1 studies [15]. Given that proportionality is part of the binding domain in MFQ-2, we expected its age association to be comparable in magnitude to the other binding foundations. Regarding gender, we predicted women would score higher on the individualizing foundations (care and equality) and purity, while men would show higher scores on the binding foundations (proportionality, loyalty, and authority), consistent with prior MFQ-1 results and with Atari, Haidt (7) who reported a modest male advantage in MFQ-2 proportionality. We further expected a male advantage in proportionality because it aligns more closely with equality- or merit-based allocation; across economic-game paradigms, men tend to endorse merit more and women equality more [20, 21].

Table 2. Gender differences in MFQ-1 moral foundations across the previous studies.

		Foundation					
Study	N	Care	Fairness	Proportionality	Loyalty	Authority	Purity
Milojev et al. (2014)	3,635	.25	.07		-.07	-.09	-.02
Atari et al. (2020), study 1	348,660	.45	.17		.03	.00	.20
Atari et al. (2020), study 2	11,969	.27	.15		-.05	-.03	.11
Nilsson (2023)	2,282	.21	.11		-.03	-.06	.02
Van Leeuwen et al. (2017)	2,478	.20	.08		-.04	.00	.10
Atari et al. (2023; MFQ-2)	3,902	.03	.16	-.09	-.06	-.06	.09
**Average**		.23	.12	-.09	-.04	-.04	.08

Note: Positive values indicate higher scores for women. All referenced studies used the MFQ-1 questionnaire, except for Atari et al. (2023), which used the updated MFQ-2.

Method

Participants

The present research draws upon two samples collected by Zakharin and Bates (10) and Zakharin and Bates (22). The samples were collected using Prolific Academic, a widely used online platform for scientific research. The first sample consisted of 1,659 UK participants (825 females, 827 males, 7 other) with a mean age of 41.16 years (SD = 13.97). The second sample consisted of 830 US participants (415 females, 415 males), with a mean age of 39.42 years (SD = 14.90).

The present study is a secondary analysis of previously collected data that are publicly available in de-identified form. The original data collection was approved by the Psychology Research Ethics Committee at the University of Edinburgh. Informed consent was obtained from all participants electronically prior to data collection. The current analyses used only anonymized, publicly shared data and therefore did not involve additional participant contact or collection of identifiable information.

Materials

Moral foundations were assessed with the MFQ-2 [7]. This instrument consists of 36 items, six for each foundation, with responses on a 5-point Likert scale ranging from 1: Does not describe me at all to 5: Describes me extremely well. Participants rate how well each statement describes them or their opinions.

Procedure

Participants from both samples completed MFQ-2 online via a Qualtrics survey. To ensure the privacy of participants, all collected data were de-identified using anonymous codes, and no personally identifying information was obtained. The authors did not have access to any information that could identify individual participants during or after data collection. Informed consent was obtained from all participants. Subjects then completed the items. A small monetary compensation was provided through the Prolific Academic platform. All statistical analyses were completed in R [23] and *umx* [24].

Results

Distributions of moral foundations' scores

We first examined the distribution of scores in each MFQ-2 foundation. For each foundation (and the binding/individualizing composites), we summarized score distributions using the mean and SD and quantified distributional shape using skewness and kurtosis. We also inspected histograms to identify clustering near the minimum or maximum response options (floor/ceiling effects). As seen in **Table** 3 and graphically in Fig 1, care and purity demonstrated the most pronounced skewness, with care showing a strong right-skew while purity showed a strong left-skew. Histograms for all remaining foundations are presented in the supplementary materials. This observation suggests the presence of potential ceiling effects for care and floor effects for purity. For completeness, we also report kurtosis. All moral foundations except care showed a platykurtic distribution characterized by fewer scores falling close to the mean and thinner tails compared to a normal distribution.

Table 3. Descriptive statistics for each moral foundation and the broad binding and individualizing domains for US and UK samples separately.

Foundation	Mean		SD		Skew		Kurtosis	
	US	UK	US	UK	US	UK	US	UK
Care	4.05	3.98	0.83	0.78	-0.84	-0.80	0.26	0.58
Equality	2.88	2.94	1.09	0.97	0.09	0.03	-0.98	-0.77
Proportionality	3.63	3.63	0.78	0.74	-0.37	-0.36	-0.31	-0.19
Loyalty	2.81	2.88	0.99	0.93	0.34	0.12	-0.74	-0.71
Authority	3.01	3.16	1.02	0.95	0.15	-0.15	-0.9	-0.76
Purity	2.26	2.20	0.97	0.87	0.72	0.73	-0.42	-0.18
Binding	2.93	2.97	0.79	0.72	0.29	0.06	-0.66	-0.58
Individualizing	3.46	3.46	0.81	0.74	-0.19	-0.25	-0.53	-0.25

Fig 1. Distribution of scores for care and purity moral foundations across both datasets

Age and Gender effects on moral foundations scores

Next, we examined the effects of age, testing the hypothesis that scores on all moral foundations would increase with age, as reported by Castilla-Estévez and Blázquez-Rincón (15). In linear regression models with age predicting each moral foundation in turn (see **Table 4**), significant increases with age were found for the binding domain and for each of its four constituent foundations in both samples, except for proportionality, which showed the expected positive relationship with age in the US but not in the UK sample. Contrary to expectation, significant reductions in scores were obtained with age for individualizing and its constituent equality concern foundation. Scores on the care foundation showed no significant association with age. The trendlines for age and scores across all six moral foundations for the US and UK samples are shown in Fig 2.

Fig 2. Relationship of foundation scores with age in the US and UK samples.

Regarding gender differences, as predicted, women scored significantly higher than men on individualizing foundations in both samples (see Table 4). The expected female superiority for purity, however, was not found. Instead, no significant gender differences were observed for purity in either sample. For loyalty and authority, the predicted higher scores for males were found in both samples. Based on the expectation for loyalty and authority, we had predicted that proportionality would also show a male advantage, which was supported (see Table 4). Given the number of comparisons across six moral foundations, two predictors (age, gender), and two samples, we prioritized the magnitude and consistency of standardized coefficients (β) over reliance on individual p-values. Nonetheless, we note that the majority of primary effects reported below survive a Bonferroni-corrected threshold of $\alpha = .05/12 = .004$ per sample, and effects that replicated across both samples provide stronger evidence than any single test.

Table 4. Standardized effects of age and sex on moral foundations scores in the US and UK samples.

Foundation	Age		Sex	
	US	UK	US	UK
**Individualizing**	-.08*	-.08***	-.19***	-.15***
Care	.05	-.04	-.17***	-.16***
Equality	-.15***	-.09***	-.16***	-.09***
**Binding**	.26***	.14***	.11***	.06*
Proportionality	.12***	.01	.14***	.10*
Loyalty	.28***	.18***	.13***	.07**
Authority	.23***	.17***	.13***	.06*
Purity	.21***	.07**	-.01	-.02

Note: For sex effects, positive β values reflect higher scores for males relative to females. Significance levels are indicated as follows: ***p < .001, **p < .01, *p < .05.

Discussion

Our study revealed significant age and gender differences in moral foundations, along with notable response distributions for care and purity. Care showed a ceiling effect with modal responses clustered near the maximum possible score, while purity displayed a floor effect with modal responses near the minimum. Examining age effects, we found increases in all binding foundations and decreases in support for equality with age, while care scores remained stable across the age range. Regarding the effects of gender, as predicted, women scored higher on care and equality and lower on three out of four binding foundations (authority, loyalty, proportionality). Contrary to the prediction, no gender differences in purity were found.

Comparing our results to those based on the MFQ-1 [15], average effect sizes in our study were generally larger, perhaps suggesting that the improved measurement characteristics of the MFQ-2 enhanced its sensitivity. Specifically, correlations between age and binding foundations averaged approximately .16 in our current MFQ-2 data, compared to .06 to .08 in the meta-analysis of MFQ-1 data. The directions of the effects, however, differed considerably in places from what had been observed for the MFQ-1.

The MFQ-1 and 2 differ most clearly in their treatment of fairness, with the MFQ-2 breaking the old fairness foundation into two independent dimensions of equality and proportionality. Whereas Castilla-Estévez and Blázquez-Rincón (15) found that scores on the old fairness domain increased with age, for the MFQ-2-based data in the present study, proportionality increased, but equality scores *decreased* with age, confirming Atari, Haidt (7) findings. This difference in sign of the age effects on equality and proportionality previously combined into a single foundation supports the more refined two-factor model of fairness provided by the MFQ-2. Turning to the second individualizing foundation, care, no significant effects of age were found, and the small differences observed oscillated around zero, with opposite sign in the two samples, suggesting that this foundation is unaffected by age.

While the effects of age on binding foundations may appear modest (r ~ .18), it is important to place them in the context of the long human lifespan where their practical significance becomes apparent. Each person transits through ages – at one time, we are infants, teens, and, hopefully, make our three score and ten or more. Across this wide range, even small annual changes lead to large cumulative gaps. For example, the expected difference in binding foundation scores between an 18-year-old and an 80-year-old was 0.78 SD in our sample. These differences, accumulated over decades, thus result in substantially different moral worldviews between younger and older adults.

These divergences have tangible societal implications. The sharp age-related decline in Equality concerns, combined with the rise in Binding foundations, suggests a mechanism for the well-documented conservative shift associated with aging. Furthermore, the 0.78 SD gap in binding scores between the youngest and oldest cohorts provides a quantitative basis for current intergenerational conflicts ('culture wars'). Where younger cohorts may view the prioritization of tradition and authority as inherently oppressive, older cohorts may view the dismissal of these foundations as a failure of moral duty. Such foundation-level disconnects complicate cross-generational political consensus. These findings are consistent with binding foundations reinforcing group loyalty, respect for authority, and traditional values. As individuals accumulate life experience, they may develop a greater appreciation for the role of authority, tradition, and established social hierarchies in maintaining a well-functioning society, leading to stronger endorsement of binding foundations.

Regarding gender disparities, the observed pattern aligns well with previous findings of higher individualizing and lower binding scores in women found in studies using the MFQ-1 [e.g. 18]. However, unlike previous studies that reported women scoring higher than men on purity, we found no evidence of gender differences here. This discrepancy may be explained by

improvements in the measurement of purity foundation in MFQ-2, which excludes the disgust-related items present in MFQ-1. On this view, the earlier gender differences may have reflected women's higher disgust sensitivity, a well-known effect [25, 26]. Thus, women tended to prioritize care, egalitarian outcomes and oppose meritocratic or proportional division, while men placed greater weight than women on loyalty, obedience to authority, and meritocratic forms of fairness. While modest in size, these gender differences, coupled with significant changes in gender roles in society, may have had a significant contribution to societal shifts toward more egalitarian and less hierarchical forms of governance.

These findings underscore the importance of considering both maturational and societal drivers. Maturational mechanisms likely include 'social investment' processes, where taking on adult roles (parenthood, employment) necessitates increased valuation of stability, authority, and loyalty. Simultaneously, societal drivers likely reflect the 'Flynn effect for morality' or broad secular liberalization, pushing cohort means toward higher Care and lower Purity. Given their shared cultural and economic structures, we expect these dual pressures to operate similarly across US and UK contexts, though their relative strength may vary with national factors such as religiosity and political culture.

Finally, in the results of distribution analyses, we found clear evidence of floor and ceiling effects. As shown in Fig 1, care and purity showed almost mirror-image shifts – with care having a modal response near the ceiling, while for purity, the modal response was almost the lowest possible score on the scale. Thus, in addition to age effects on binding foundations and reduced concerns for equality with cumulative effects nearing 1SD across the lifespan, we find evidence for population-level shifts of moral foundations scores downward for purity and upward for care.

Limitations and future directions

Several limitations of this study suggest promising directions for future research. First, the associations we report are between-person age differences and cannot be taken as evidence of within-person developmental (maturational) change. Age is confounded with birth cohort and historical period: older participants differ not only in age but also in the social and cultural context in which they were raised. Thus, the observed age-related increase in binding foundations (and decrease in equality) could reflect maturation, cohort-based socialization, or both. Longitudinal—ideally cohort-sequential—data are needed to separate age, cohort, and period influences. Second, while self-report measures like the MFQ-2 have established utility, incorporating behavioral measures of moral and ethical decision-making would provide valuable complementary evidence.

Finally, our focus on US and UK samples limits generalizability, as cultures imbue moral values with different significance [7, 13]. Future work should examine these patterns in diverse cultural contexts. Additionally, the observed floor and ceiling effects in purity and care scores may reflect broader societal influences that deserve systematic investigation, including socioeconomic factors, educational trends [27, 28], and religious engagement. Historiographic approaches could help illuminate these sociocultural dynamics, for instance by analyzing moral themes in historical literature and other cultural artifacts [29].

Conclusion

In conclusion, in two studies, we found increases in all binding foundations and decreases in support for equality with age, while care scores remained stable across the age range. For gender, women, as predicted, scored higher on both care and equality and lower on three out of four binding foundations (authority, loyalty, proportionality). Contrary to the prediction, no gender differences in purity were found. These findings underscore the importance of considering maturational [30, 31] and societal [32] drivers of moral values.

Data Accessibility Statement

The data and materials used in this paper, as well as the R code used to generate the results, are openly available at the OSF site for this paper at https://osf.io/yk3qc/?view_only=38928cc2387d489cbb22039dc4ecb159

References

1. Freeman KJ. Schools of hellas: an essay on the practice and theory of ancient Greek education from 600 to 300 BC: Macmillan; 1912.
2. Costanza DP, Rudolph CW, Zacher H. Are generations a useful concept? Acta Psychol (Amst). 2023;241:104059. Epub 20231021. doi: 10.1016/j.actpsy.2023.104059. PubMed PMID: 37871402.
3. Erikson EH, Erikson JM, Kivnick HQ. Vital involvement in old age. New York: WW Norton & Company; 1994.
4. Kohlberg L, Kramer R. Continuities and discontinuities in childhood and adult moral development. HUM DEV. 1969;12(2):93-120. doi: 10.1159/000270857. PubMed PMID: 5791921.
5. Haidt J, Joseph C. Intuitive ethics: How innately prepared intuitions generate culturally variable virtues. Daedalus. 2004;133(4):55-66.
6. Graham J, Nosek BA, Haidt J, Iyer R, Koleva S, Ditto PH. Mapping the moral domain. J Pers Soc Psychol. 2011;101(2):366-85. Epub 2011/01/20. doi: 10.1037/a0021847. PubMed PMID: 21244182; PubMed Central PMCID: PMCPMC3116962.
7. Atari M, Haidt J, Graham J, Koleva S, Stevens ST, Dehghani M. Morality beyond the WEIRD: How the nomological network of morality varies across cultures. Journal of Personality and Social Psychology. 2023.
8. Baumard N, André J-B, Sperber D. A mutualistic approach to morality: The evolution of fairness by partner choice. Behavioral and Brain Sciences. 2013;36(1):59-78.
9. Lin C-A, Bates TC. Free to choose: Mutualist motives for partner choice, proportional division, punishment, and help. Plos one. 2022;17(5):e0266735.
10. Zakharin M, Bates TC. Moral Foundations Theory: Validation and replication of the MFQ-2. Personality and Individual Differences. 2023;214:112339.
11. Davies CL, Sibley CG, Liu JH. Confirmatory Factor Analysis of the Moral Foundations Questionnaire. Social Psychology. 2014;45(6):431-6. doi: 10.1027/1864-9335/a000201.
12. Doğruyol B, Alper S, Yilmaz O. The five-factor model of the moral foundations theory is stable across WEIRD and non-WEIRD cultures. Personality and Individual Differences. 2019;151. doi: 10.1016/j.paid.2019.109547.
13. Iurino K, Saucier G. Testing Measurement Invariance of the Moral Foundations Questionnaire Across 27 Countries. Assessment. 2018:1073191118817916. Epub 2019/01/01. doi: 10.1177/1073191118817916. PubMed PMID: 30596252.
14. Kivikangas JM, Lönnqvist J-E, Ravaja N. Relationship of Moral Foundations to Political Liberalism-Conservatism and Left-Right Orientation in a Finnish Representative Sample. Social Psychology. 2017;48(4):246-51. doi: 10.1027/1864-9335/a000297.
15. Castilla-Estévez D, Blázquez-Rincón D. Age and moral foundations: A meta-analytic approach. The Spanish Journal of Psychology. 2021;24:e41.
16. Nilsson A. Measurement invariance of moral foundations across population strata. Journal of Personality Assessment. 2023;105(2):163-73.
17. Milojev P, Osborne D, Greaves LM, Bulbulia J, Wilson MS, Davies CL, et al. Right-wing authoritarianism and social dominance orientation predict different moral signatures. Social Justice Research. 2014;27:149-74.
18. Atari M, Lai MH, Dehghani M. Sex differences in moral judgements across 67 countries. Proceedings of the Royal Society B. 2020;287(1937):20201201.
19. Van Leeuwen F, Dukes A, Tybur JM, Park JH. Disgust sensitivity relates to moral foundations independent of political ideology. Evolutionary Behavioral Sciences. 2017;11(1):92.
20. Fehr E, Naef M, Schmidt KM. Inequality aversion, efficiency, and maximin preferences in simple distribution experiments: Comment. American Economic Review. 2006;96(5):1912-7.

21. Capraro V. Gender differences in the trade-off between objective equality and efficiency. Judgment and Decision Making. 2020;15(4):534-44.
22. Zakharin M, Bates TC. Higher cognitive ability linked to weaker moral foundations in UK adults. Intelligence. 2025;111:101930.
23. R Core Team. R: A language and environment for statistical computing. R Foundation for Statistical Computing, Vienna, Austria; 2023.
24. Bates TC, Maes H, Neale MC. umx: Twin and Path-Based Structural Equation Modeling in R. Twin Res Hum Genet. 2019;22(1):27-41. Epub 2019/04/05. doi: 10.1017/thg.2019.2. PubMed PMID: 30944056.
25. Tybur JM, Lieberman D, Griskevicius V. Microbes, mating, and morality: individual differences in three functional domains of disgust. Journal of personality and social psychology. 2009;97(1):103.
26. Al-Shawaf L, Lewis DM, Buss DM. Sex differences in disgust: Why are women more easily disgusted than men? Emotion review. 2018;10(2):149-60.
27. Schoon I, Cheng H, Gale CR, Batty GD, Deary IJ. Social status, cognitive ability, and educational attainment as predictors of liberal social attitudes and political trust. Intelligence. 2010;38(1):144-50.
28. Deary IJ, Batty GD, Gale CR. Bright children become enlightened adults. Psychol Sci. 2008;19(1):1-6. doi: 10.1111/j.1467-9280.2008.02036.x. PubMed PMID: 18181782.
29. Baumard N. Psychological origins of the Industrial Revolution. Behavioral and Brain Sciences. 2018;42:e189. Epub 20180411. doi: 10.1017/S0140525X1800211X. PubMed PMID: 30259818.
30. Roberts BW, Wood D, Caspi A. The development of personality traits in adulthood. 2008.
31. Lodi-Smith J, Spain SM, Cologgi K, Roberts BW. Development of identity clarity and content in adulthood. Journal of Personality and Social Psychology. 2017;112(5):755.
32. Baumard N, Safra L, Martins M, Chevallier C. Cognitive fossils: using cultural artifacts to reconstruct psychological changes throughout history. Trends Cogn Sci. 2024;28(2):172-86. Epub 20231108. doi: 10.1016/j.tics.2023.10.001. PubMed PMID: 37949792.

Fig 1. Distribution of scores for care and purity moral foundations

Click here to access/download;Figure;Fig1.tif

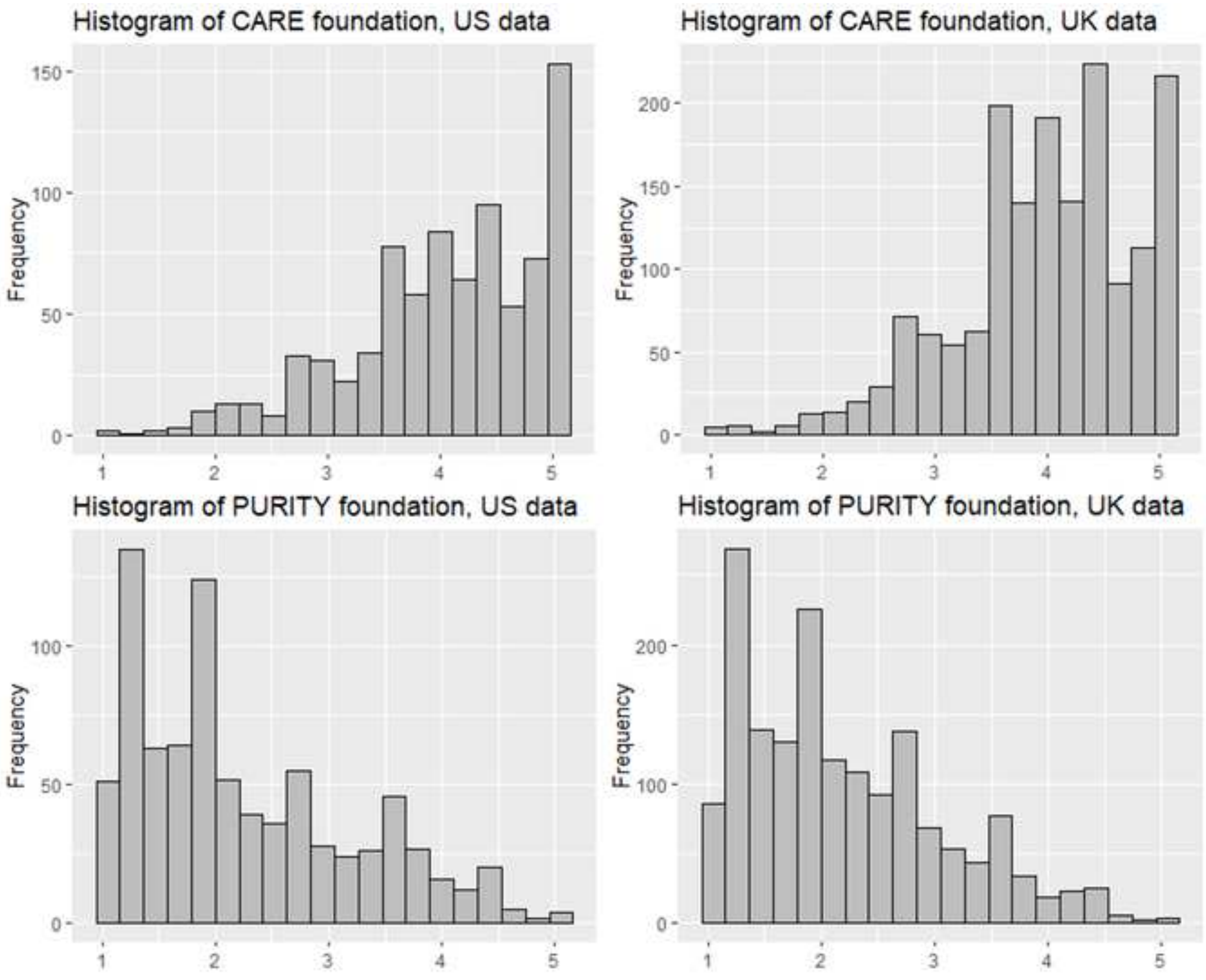

Fig 2. Relationship of foundation scores with age in the US and UK samples

Click here to access/download;Figure;Fig 2.tif

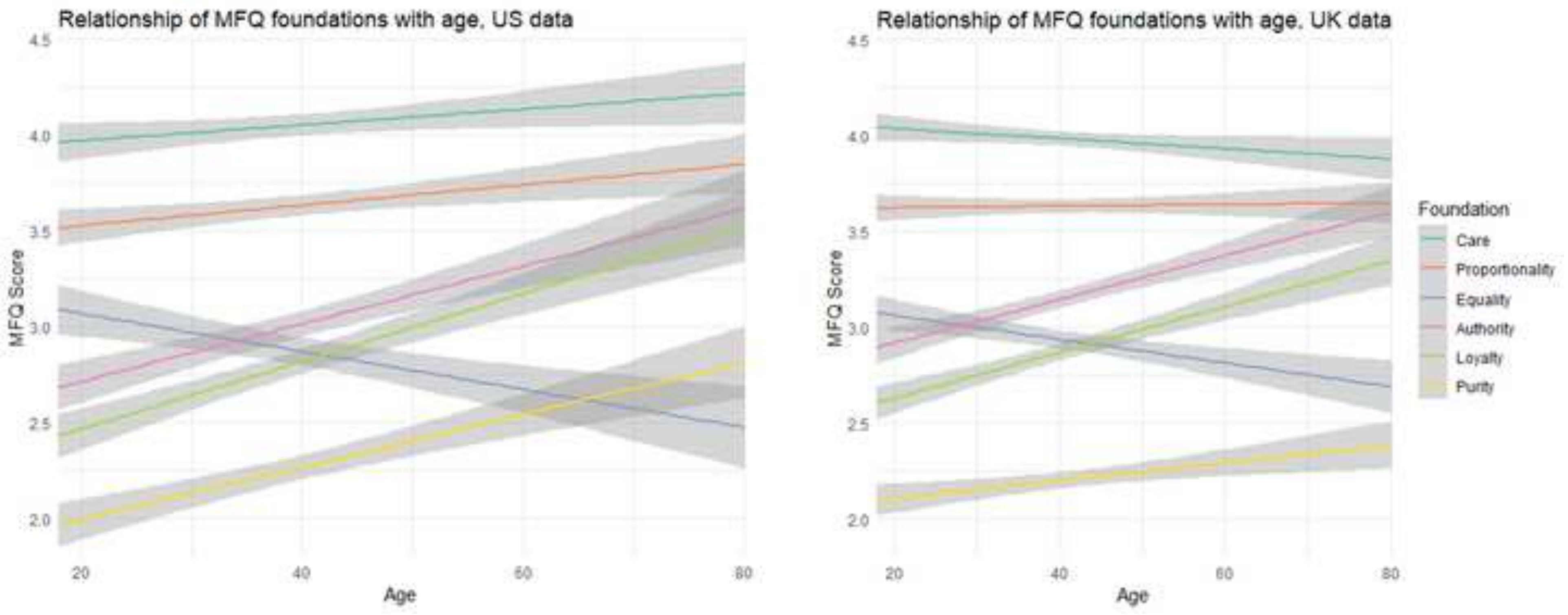

Click here to access/download

Supporting Information

S1 File. MFQ-1 Items and Domains.docx

Click here to access/download
Supporting Information
S2 File. MFQ-2 Items and Domains.docx

Revised Manuscript with Track Changes

Age, gender, and score distributions of moral foundations

Authors

Michael Zakharin and Timothy C Bates

Address

Department of Psychology, University of Edinburgh

7 George Square

Edinburgh EH8 9JZ

Telephone +44 131 650 1945

tim.bates@ed.ac.uk (Orcid: 0000-0002-1153-9007) or michael.zakharin@vcuhealth.org (Orcid: 0000-0003-3689-4635)

Abstract

Claims of generational differences in moral values have important implications for understanding societal change. Using the recently developed Moral Foundations Questionnaire (MFQ-2), we examined age and gender effects on moral foundations in large US (N = 835) and UK (N = 1,659) samples. Across both nations, binding foundations (proportionality, loyalty, authority and purity) showed consistent age-related increases. While these yearly increments were modest, they accumulated to substantial differences between youngest and oldest participants, with binding scores approximately 0.75 standard deviations higher in older adults. Among individualizing foundations, the preference for equality decreased with age, while harm/care remained stable. Gender differences emerged systematically: women scored higher on individualizing foundations, while men showed elevated scores on binding foundations. We also observed significant distributional effects, with care scores clustering near the scale maximum and purity scores near the minimum. These demographic patterns suggest important dynamics in how moral values may shift across generations and between genders, with implications for understanding social change.

Introduction

"*Children began to be the tyrants, not the slaves, of their households. They no longer rose from their seats when an elder entered the room; they contradicted their parents, chattered before company, gobbled up the dainties at table, and committed various offences against Hellenic tastes, such as crossing their legs. They tyrannised over the paidagogoi and schoolmasters*" (Freeman, 1912, p. 74).

As noted by Freeman (1912), ancient writers, particularly those from ancient Greece such as Aristophanes and Xenophon, argued that the young of their day lacked the moral rectitude they themselves had shown at their age. These persistent claims of generational moral differences warrant rigorous investigation, as documenting either the presence or absence of age-related variations in moral foundations carries significant implications for understanding societal changes. Differences between young and old, of course, may refer to very different phenomena and causes (Costanza et al., 2023). One possibility is that moral foundations exhibit reliable developmental change, as suggested by classic theories of moral development, which propose a stage-like process of increasing moral concern (Erikson et al., 1994; Kohlberg & Kramer, 1969). Alternatively, change may affect the culture as a whole, impacting persons of all ages and manifesting as a shift in the mean score for the population. In such mean-shift cases, older generations who lived through an earlier, perhaps stricter (or more lax) time will observe that young people differ not only from their own youth but potentially from how their parents were at the same age. Here, we use the Moral Foundations Questionnaire- 2 (MFQ-2) to examine differences in the moral foundations in two new samples, one US and one UK, to describe mean differences in moral foundations by age and gender and the shape of MFQ-2 scores in the population—specifically examining distributional properties such as skew and ceiling/floor effects—and the changes exhibited with age.to give a picture of both the shape of moral foundations—specifically examining distributional properties such as skew and ceiling/floor effects—and the changes exhibited with ageto give a picture of both the shape of moral foundations—their distribution in the population—and the changes, if any, exhibited with age, as well as effects of gender. In this paper, we use distributional effects to mean features of the score distributions beyond mean differences, such as skewness/kurtosis and clustering near the scale endpoints (ceiling/floor effects), which can indicate population-level shifts in how moral concerns are expressed. We first background moral foundation theory and work on age and gender differences in foundation scores.

Moral foundations theory (MFT; Atari et al., 2023; Graham et al., 2011; Haidt & Joseph, 2004) proposes that moral judgment is supported by multiple partially distinct concerns rather than

a single moral principle. In this paper we focus on the six foundations assessed by the MFQ-2: care, equality, proportionality, loyalty, authority, and purity. Care captures concern for others' suffering and harm prevention. Equality reflects egalitarian concern that people should be treated similarly and have similar outcomes. Proportionality reflects merit-based fairness - benefits and punishments should track effort, contribution, or responsibilityoffers a comprehensive framework for moral judgment. Rather than focusing on a single or overarching moral process, MFT suggests that moral decision-making is pluralistic – incorporating at least six distinct foundational domains underpinning moral judgment: care, equality, proportionality, loyalty, authority and purity, as well as liberty (Iyer et al., 2012; Zakharin & Bates, 2024b). Within MFT, each foundation constitutes a distinct aspect of moral judgment. The MFQ-1 (Graham et al., 2011) identified five core moral foundations. The *care* foundation captures concern for the well-being of others and a motive for harm prevention (Amormino et al., 2022; Nilsson et al., 2020). Those who strongly endorse this foundation tend to value kindness, compassion, and nurturance (Feldman, 2021). The *fairness* foundation centres on preventing unfair or unequal treatment of others (Vecina & Piñuela, 2017), as well as ensuring reciprocity (Trivers, 1971). When activated, this foundation elicits anger if one is directly subjected to unfair actions or exploitation or if another person is the target of such actions (Gao et al., 2016). The *loyalty* foundation emphasizes loyalty to one's group or nation (Prince et al., 2019). High scores on this foundation have been linked to patriotism, self-sacrifice, and not betraying one's group (Clifford, 2017; Graham et al., 2009). The *authority* foundation emphasizes respect for legitimate authority, tradition and order, deference, and fulfilling duties (Harnish et al., 2018; Milojev et al., 2014). Finally, the *purity* foundation focuses on the importance of bodily and spiritual cleanliness (Paruzel-Czachura et al., 2023; Selterman & Koleva, 2015; Van Tongeren et al., 2021) and control of appetites (Fitouchi et al., 2023). Conceptually, the foundations are grouped into two broad domains: individualizing and binding. The individualizing domain encompasses care and fairness and focuses on the concern for individual well-being and rights (Graham et al., 2011). In contrast, the binding domain includes loyalty, authority and purity, emphasising the group's welfare and cohesion. While this higher-order clustering of foundations has been supported in Western cultures, its robustness in non-Western countries may be less clear (Atari et al., 2023).

Moral foundations are assessed using the Moral Foundations Questionnaire (MFQ-1; Graham et al., 2011) and, most recently, the Moral Foundations Questionnaire-2 (MFQ-2: Atari et al., 2023). The MFQ-2 differs from the MFQ-1 in several notable ways. First, where the MFQ-1 had a single fairness foundation, the MFQ-2 separated this into independent foundations of equality and proportionality. Equality measures egalitarian concern for equal outcomes (Wolff, 1998), while proportionality, similar to the concept of mutualism (conceptually related to mutualism; Baumard

et al., 2013; Lin & Bates, 2022).~~,~~ Loyalty concerns commitment to one's group and aversion to betrayal. Authority concerns respect for legitimate hierarchy, tradition, and role-based duties. Purity concerns contamination and sanctity, including bodily and spiritual cleanliness and self-control. These foundations are often summarized into higher-order clusters in Western samples, with individualizing concerns (care, equality) emphasizing protection and equal regard for individuals, and binding concerns (loyalty, authority, purity, and proportionality) emphasizing group cohesion, order, and norm enforcement. (Atari et al., 2023; Graham et al., 2011).

Moral foundations are commonly measured with the MFQ-1 (Graham et al., 2011) and the updated MFQ-2 (Atari et al., 2023). The key conceptual difference is that MFQ-1 assesses five foundations, including a single fairness scale, whereas recently developed MFQ-2 assesses six foundations by splitting fairness into two separable constructs: equality and proportionality. In addition, MFQ-2 replaces the original MFQ-1 items with newly written or rephrased items, intended to improve clarity and content coverage and to better recover the expected factor structure in contemporary samples (Atari et al., 2023; Zakharin & Bates, 2023), addressing well-documented psychometric limitations of MFQ-1 in some datasets (e.g. Davies et al., 2014; Doğruyol et al., 2019; Iurino & Saucier, 2018; Kivikangas et al., 2017). The complete item sets for MFQ-1 and MFQ-2 are provided in the Supporting Information ~~measures a motive to ensure that rewards and punishments are proportional to contributions. Second, all item content was revised with newly written or rephrased content. This revision improved the clarity and specificity of the items and enhanced the comprehensiveness of the scales - the new instrument exhibits excellent statistical properties and matches its expected factorial structure well (Atari et al., 2023; Zakharin & Bates, 2023) compared to the MFQ-1 (Davies et al., 2014; Doğruyol et al., 2019; Harper & Rhodes, 2021; Iurino & Saucier, 2018; Kivikangas et al., 2017; Nilsson & Erlandsson, 2015; Yalçındağ et al., 2017; Zakharin & Bates, 2021). The full item sets for MFQ-1 and MFQ-2 are available as a supporting Information file.~~.

Having described the MFT foundations and measures, we next turn to existing research on the relationships of age and sex to moral foundations, focussing on studies with large sample sizes that offer the most relevant and robust findings.

Age differences in moral foundations scores

The most comprehensive examination of age effects on moral foundations scores is the meta-analysis of 239 independent samples and 492,298 subjects using the MFQ-1, as reported by Castilla-Estévez and Blázquez-Rincón (2021). Their analysis revealed a consistent albeit small (mean $\beta = 0.06$) age-related increase in moral concern across all foundations (see Table 1). Two

Formatted: Font: (Default) Times New Roman

Formatted: Left

studies not included in Castilla-Estévez and Blázquez-Rincón (2021) support this finding, with Nilsson (2023) replicating the increase in all foundations in a Swedish sample (N = 2,282), with around twice the effect size as reported by Castilla-Estévez and Blázquez-Rincón (2021). In a New Zealand sample, Milojev et al. (2014) also found that scores on all foundations increased with age, with a mean age effect of .21 and larger effects in binding than individualizing. These studies and their estimates of age effects on moral foundations are summarized in Table 1. While these effects may appear modest, it is important to note that small increases, when accumulated over many decades of life, result in substantial differences in scores between the youngest and oldest members of the population.

Table 1

Correlations between MFQ-1 moral foundations and age across the previous studies.

	Individualising			Binding		
Study	Care	Fairness/ Equality	Proport ionality	Loyalty	Authority	Purity
Castilla-Estévez & Blázquez-Rincón (2021) N = 492,298	.08	.06	--	.04	.06	.07
Nilsson (2023) N = 2,282 (Swedish)	.13	.07	--	.18	.07	.13
Milojev et al. (2014) N = 3635 (New Zealand)	.05	.15	--	.26	.29	.29
Atari et al. (2023; MFQ-2) N = 1410	.09	-.08	.04	.11	.12	.09

Gender differences in moral foundations scores

The most extensive study of gender differences in MFQ-1 scores comes from Study 1 of Atari et al. (2020), reporting data from the online international dataset hosted on YourMorals.org (N = 348,660, with 67 countries represented with the majority (N = 262,629) coming from the US). Atari et al. (2020) reported higher scores in women for care, fairness, and purity, with care showing the greatest disparity. However, no significant differences were observed for loyalty or authority. These patterns were confirmed in an independent international sample (N = 11,969), although the gender gap in care scores was narrower, more closely matching the differences observed in fairness and purity (see Table 2). Similar results were also reported by Van Leeuwen et al. (2017) using an international sample primarily from Western countries (N = 2,478), see Table 2. Again, women scored higher on care, fairness, and purity, and men scored higher on loyalty, with no difference found in authority. Likewise, in a Swedish sample (N = 2282: Nilsson, 2023), gender differences in care and fairness were replicated alongside a slight male advantage in authority, though the gender effect on purity did not hold. Finally, a study from New Zealand (N = 3,635) by Milojev et al. (2014) echoed the gender discrepancies in care and fairness and found modest but significant gender differences in the binding foundations favouring men, with no significant effects found for purity.

The introduction of the updated MFQ-2 (Atari et al., 2023) provides an opportunity to reassess these relationships with a more refined instrument. This study, therefore, aimed to verify these findings with the new measure. Currently, only one study has investigated age and gender differences with MFQ-2 (Atari et al., 2023). This study confirmed positive associations between age and all moral foundations except equality and higher scores for women in fairness (MFQ-2 equality) and purity (see Tables 1 and 2). Men scored modestly higher in loyalty, authority, and the newly added proportionality foundation. Notably, gender differences in care were minimal.

Drawing on these MFQ-2 findings and the broader MFQ-1 literature, we hypothesized that our study would show age-related increases across all foundations (effect sizes ranging from .05 to .25), except possibly equality, consistent with the distribution of age effects reported in large-scale MFQ-1 studies (Castilla-Estévez & Blázquez-Rincón, 2021). Given that proportionality is part of the binding domain in MFQ-2, we expected its age association to be comparable in magnitude to the other binding foundationsWe expected this pattern would extend to the new proportionality foundation. Regarding gender, we predicted women would score higher on the individualizing foundations (care and equality) and purity, while men would show higher scores on the binding foundations (-of proportionality, loyalty, and authority), consistent with prior MFQ-1 results and with Atari et al. (2023) who reported a modest male advantage in MFQ-2 proportionality. We further expected a male advantage in proportionality because it aligns more closely with equality- or merit-based allocation; across economic-game paradigms, men tend to endorse merit more and women equality more (Capraro, 2020; Fehr et al., 2006).

Table 2

Gender differences in MFQ-1 moral foundations across the previous studies.

		Foundation					
Study	N	Care	Fairness	Proportionality	Loyalty	Authority	Purity
Milojev et al. (2014)	3,635	.25	.07		-.07	-.09	-.02
Atari et al. (2020), study 1	348,660	.45	.17		.03	.00	.20
Atari et al. (2020), study 2	11,969	.27	.15		-.05	-.03	.11
Nilsson (2023)	2,282	.21	.11		-.03	-.06	.02
Van Leeuwen et al. (2017)	2,478	.20	.08		-.04	.00	.10
Atari et al. (2023; MFQ-2)	3,902	.03	.16	-.09	-.06	-.06	.09
	**Average**	.23	.12	-.09	-.04	-.04	.08

Note: Positive values indicate higher scores for women. All referenced studies used the MFQ-1 questionnaire, except for Atari et al. (2023), which used the updated MFQ-2.

Method

Participants

The present research draws upon two samples collected by Zakharin and Bates (2023) and Zakharin and Bates (2024a). The samples were collected using Prolific Academic, a widely used online platform for scientific research. The first sample consisted of 1,659 UK participants (825 females, 827 males, 7 other) with a mean age of 41.16 years (SD = 13.97). The second sample consisted of 830 US participants (415 females, 415 males), with a mean age of 39.42 years (SD = 14.90).

Materials

Moral foundations were assessed with the MFQ-2 (Atari et al., 2023). This instrument consists of 36 items, six for each foundation, with responses on a 5-point Likert scale ranging from 1: Does not describe me at all to 5: Describes me extremely well. Participants rate how well each statement describes them or their opinions.

Procedure

Participants from both samples completed MFQ-2 online via a Qualtrics survey. To ensure the privacy of participants, all collected data were de-identified using anonymous codes, and no personally identifying information was obtained. The authors did not have access to any information that could identify individual participants during or after data collection. Informed consent was obtained from all participants. Subjects then completed the items. A small monetary compensation was provided through the Prolific Academic platform. All statistical analyses were completed in R (R Core Team, 2023) and *umx* (Bates et al., 2019).

Results

Distributions of moral foundations' scores

We first examined the distribution of scores in each MFQ-2 foundation. For each foundation (and the binding/individualizing composites), we summarized score distributions using the mean and SD and quantified distributional shape using skewness and kurtosis. We also inspected histograms to identify clustering near the minimum or maximum response options (floor/ceiling effects)~~Our primary interest was in seeking evidence of mean shifts in the form of~~

~~skewness of score toward either the lower or upper bound of the scale~~. As seen in **Table** 3 and graphically in Figure 1, care and purity demonstrated the most pronounced ~~significant~~ skewness, with care showing a strong left-skew while purity showed a strong right-skew. Histograms for all remaining foundations are presented in the supplementary materials. This observation suggests the presence of potential ceiling effects for care and floor effects for purity. For completeness, we also report kurtosis. All moral foundations except care showed a platykurtic distribution characterized by fewer scores falling close to the mean and thinner tails compared to a normal distribution.

Table 3

Descriptive statistics for each moral foundation and the broad binding and individualizing domains for US and UK samples separately.

Foundation	Mean		SD		Skew		Kurtosis	
	US	UK	US	UK	US	UK	US	UK
Care	4.05	3.98	0.83	0.78	-0.84	-0.80	0.26	0.58
Equality	2.88	2.94	1.09	0.97	0.09	0.03	-0.98	-0.77
Proportionality	3.63	3.63	0.78	0.74	-0.37	-0.36	-0.31	-0.19
Loyalty	2.81	2.88	0.99	0.93	0.34	0.12	-0.74	-0.71
Authority	3.01	3.16	1.02	0.95	0.15	-0.15	-0.9	-0.76
Purity	2.26	2.20	0.97	0.87	0.72	0.73	-0.42	-0.18
Binding	2.93	2.97	0.79	0.72	0.29	0.06	-0.66	-0.58
Individualising	3.46	3.46	0.81	0.74	-0.19	-0.25	-0.53	-0.25

Figure 1

Distribution of scores for care and purity moral foundations across both datasets

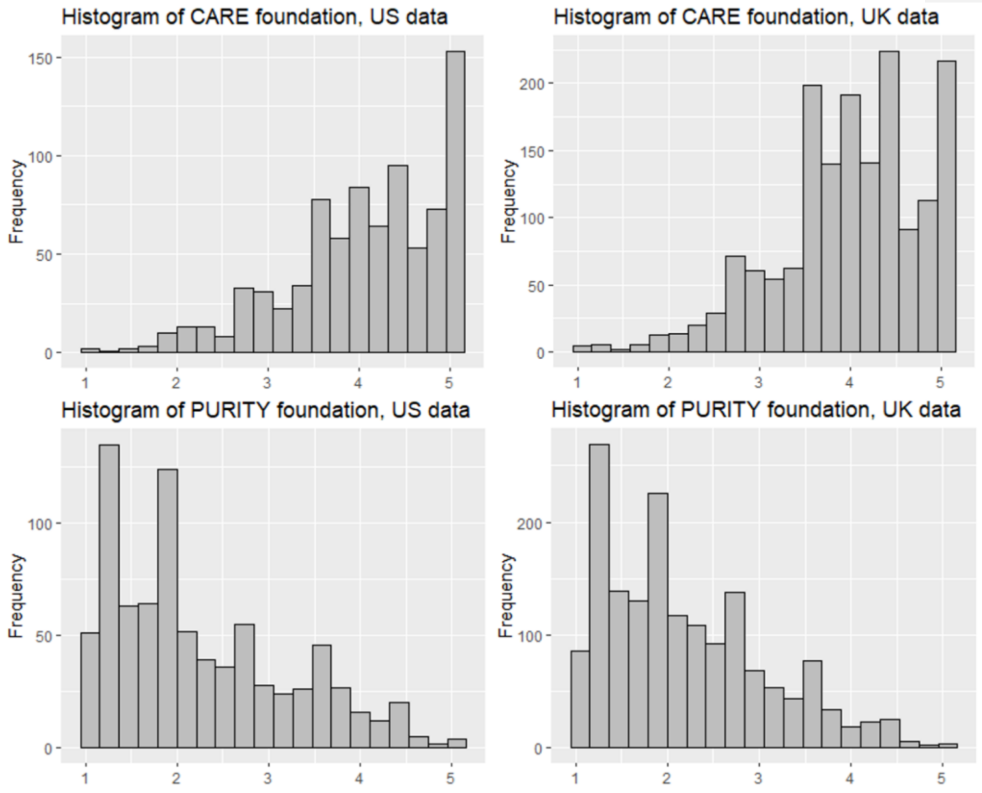

Age and Gender effects on moral foundations scores

Next, we examined the effects of age, testing the hypothesis that scores on all moral foundations would increase with age, as reported by Castilla-Estévez and Blázquez-Rincón (2021). In linear regression models with age predicting each moral foundation in turn (see **Table 4**), significant increases with age were found for the binding domain and for each of its four constituent foundations in both samples, except for proportionality, which showed the expected positive relationship with age in the US but not in the UK sample. Contrary to expectation, significant reductions in scores were obtained with age for individualizing and its constituent equality concern foundation. Scores on the care foundation showed no significant association with age. The trendlines for age and scores across all six moral foundations for the US and UK samples are shown in Figure 2.

Regarding gender differences, as predicted, women scored significantly higher than men on individualizing foundations in both samples (see Table 4). The expected female superiority for purity, however, was not found. Instead, no significant gender differences were observed for purity in either sample. For loyalty and authority, the predicted higher scores for males were found in both samples. ~~The proportionality foundation is new to the MFQ-2.~~ Based on the expectation for loyalty and authority, we had predicted that proportionality would also show a male advantage, which was supported (see Table 4). Given the number of comparisons across six moral foundations, two predictors (age, gender), and two samples, we prioritized the magnitude and consistency of standardized coefficients (β) over reliance on individual p-values. Nonetheless, we note that the majority of primary effects reported below survive a Bonferroni-corrected threshold of $\alpha = .05/12 = .004$ per sample, and effects that replicated across both samples provide stronger evidence than any single test.

Table 4

Standardized effects of age and sex on moral foundations scores in the US and UK samples.

Foundation	Age		Sex	
	US	UK	US	UK
**Individualising**	-.08*	-.08***	-.19***	-.15***
Care	.05	-.04	-.17***	-.16***
Equality	-.15***	-.09***	-.16***	-.09***
**Binding**	.26***	.14***	.11***	.06*
Proportionality	.12***	.01	.14***	.10*
Loyalty	.28***	.18***	.13***	.07**

Authority	.23***	.17***	.13***	.06*
Purity	.21***	.07**	-.01	-.02

Note: For sex effects, positive β values reflect higher scores for males relative to females. Significance levels are indicated as follows: ***p < .001, **p < .01, *p < .05.

Figure 2

Relationship of foundation scores with age in the US and UK samples.

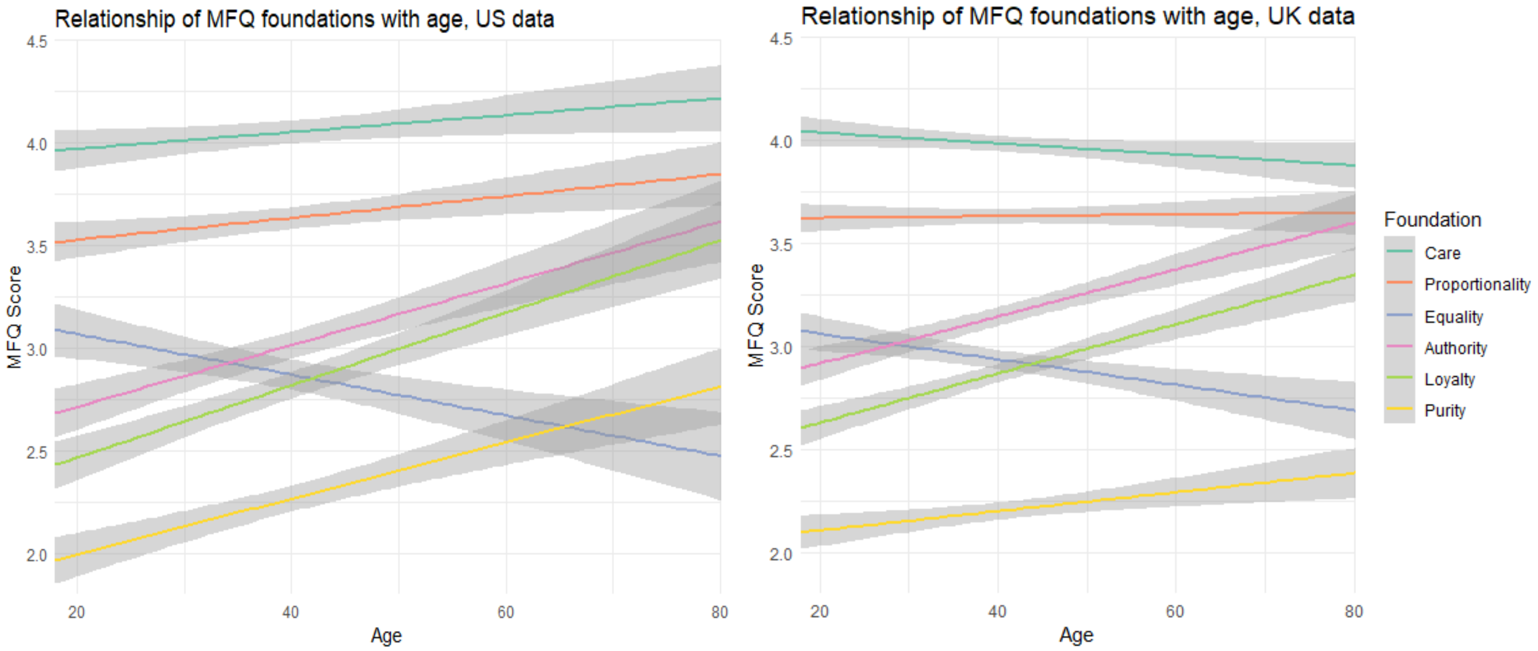

Discussion

Our study revealed significant age and gender differences in moral foundations, along with notable response distributions for care and purity. Care showed a ceiling effect with modal responses clustered near the maximum possible score, while purity displayed a floor effect with modal responses near the minimum. Examining age effects, we found increases in all binding foundations and decreases in support for equality with age, while care scores remained stable ~~was equal~~ across the age range. Regarding the effects of gender, as predicted, women scored higher on care and equality and lower on three out of four binding foundations (authority, loyalty, proportionality). Contrary to the prediction, no gender differences in purity were found.

Comparing our results to those based on the MFQ~~-~~1 (Castilla-Estévez & Blázquez-Rincón, 2021), average effect sizes in our study were generally larger, perhaps suggesting that the improved measurement characteristics of the MFQ-2 enhanced its sensitivity. Specifically, correlations between age and binding foundations averaged approximately .16 in our current MFQ-2 data, compared to .06 to .08 in the meta-analysis of MFQ-1 data. The directions of the effects, however, differed considerably in places from what had been observed for the MFQ-1.

The MFQ-1 and 2 differ most clearly in their treatment of fairness, with the MFQ-2 breaking the old fairness foundation into two independent dimensions of equality and proportionality. Whereas Castilla-Estévez and Blázquez-Rincón (2021) found that scores on the old fairness domain increased with age, for the MFQ-2-based data in the present study, proportionality increased, but equality scores *decreased* with age, confirming Atari et al. (2023) findings. This difference in sign of the age effects on equality and proportionality previously combined into a single foundation supports the more refined two-factor model of fairness provided by the MFQ-2. Turning to the second individualising foundation, care, no significant effects of age were found, and the small differences observed oscillated around zero, with opposite sign in the two samples, suggesting that this foundation is unaffected by age.

While the effects of age on binding foundations may appear modest (r ~ .18), it is important to place them in the context of the long human lifespan where their practical significance becomes apparent. Each person transits through ages – at one time, we are infants, teens, and, hopefully, make our three score and ten or more. Across this wide range, even small annual changes lead to large cumulative gaps. For example, the expected difference in binding foundation scores between an 18-year-old and an 80-year-old was 0.78 SD in our sample. These differences, accumulated over decades, thus result in substantially different moral worldviews between younger and older adults.

These divergences have tangible societal implications. The sharp age-related decline in Equality concerns, combined with the rise in Binding foundations, suggests a mechanism for the well-documented conservative shift associated with aging. Furthermore, the 0.78 SD gap in binding scores between the youngest and oldest cohorts provides a quantitative basis for current intergenerational conflicts ('culture wars'). Where younger cohorts may view the prioritization of tradition and authority as inherently oppressive, older cohorts may view the dismissal of these foundations as a failure of moral duty. Such foundation-level disconnects complicate cross-generational political consensus.

These findings are consistent with binding foundations reinforcing group loyalty, respect for authority, and traditional values. As individuals accumulate life experience, they may develop a greater appreciation for the role of authority, tradition, and established social hierarchies in maintaining a well-functioning society, leading to stronger endorsement of binding foundations.

Regarding gender disparities, the observed pattern aligns well with previous findings of higher individualising and lower binding scores in women found in studies using the MFQ-1 (e.g. Atari et al., 2020). However, unlike previous studies that reported women scoring higher than men on purity, we found no evidence of gender differences here. This discrepancy may be explained by improvements in the measurement of purity foundation in MFQ-2, which excludes the disgust-related items present in MFQ-1. On this view, the earlier gender differences may have reflected women's higher disgust sensitivity, a well-known effect (Al-Shawaf et al., 2018; Tybur et al., 2009). Thus, women tended to prioritize care, egalitarian outcomes and oppose meritocratic or proportional division, while men placed greater weight than women on loyalty, obedience to authority, and meritocratic forms of fairness. While modest in size, these gender differences, coupled with significant changes in gender roles in society, may have had a significant contribution to societal shifts toward more egalitarian and less hierarchical forms of governance.

These findings underscore the importance of considering both maturational and societal drivers. Maturational mechanisms likely include 'social investment' processes, where taking on adult roles (parenthood, employment) necessitates increased valuation of stability, authority, and loyalty. Simultaneously, societal drivers likely reflect the 'Flynn effect for morality' or broad secular liberalization, pushing cohort means toward higher Care and lower Purity. Given their shared cultural and economic structures, we expect these dual pressures to operate similarly across US and UK contexts, though their relative strength may vary with national factors such as religiosity and political culture.

Finally, in the results of distribution analyses, we found clear evidence of floor and ceiling effects. As shown in Figure 1, care and purity showed almost mirror-image shifts – with care having a modal response near the ceiling, while for purity, the modal response was almost the lowest possible score on the scale. Thus, in addition to age effects on binding foundations and reduced concerns for equality with cumulative effects nearing 1SD across the lifespan, we find evidence for population-level shifts of moral foundations scores downward for purity and upward for care.

Limitations and future directions

Several limitations of this study suggest promising directions for future research. First, the associations we report are between-person age differences and cannot be taken as evidence of within-person developmental (maturational) change. Age is confounded with birth cohort and historical period: older participants differ not only in age but also in the social and cultural context in which they were raised. Thus, the observed age-related increase in binding foundations (and decrease in equality) could reflect maturation, cohort-based socialization, or both. Longitudinal—ideally cohort-sequential—data are needed to separate age, cohort, and period influences. Second, while self-report measures like the MFQ-2 have established utility, incorporating behavioural measures of moral and ethical decision-making would provide valuable complementary evidence.

Finally, Oour focus on US and UK samples limits generalizability, as cultures imbue moral values with different significance (Atari et al., 2023; Iurino & Saucier, 2018). Future work should examine these patterns in diverse cultural contexts. Additionally, the observed floor and ceiling effects in purity and care scores may reflect broader societal influences that deserve systematic investigation, including socioeconomic factors, educational trends (Deary et al., 2008; Schoon et al., 2010), and religious engagement. Historiographic approaches could help illuminate these sociocultural dynamics, for instance by analyzing moral themes in historical literature and other cultural artifacts (Baumard, 2018).

Formatted: Left

Limitations and future directions (revised)

Several limitations of this study suggest promising directions for future research. First, the data are cross-sectional, so the observed associations with age should be interpreted as age-related differences at one point in time rather than within-person developmental change. Apparent “age effects” may reflect a mixture of maturational change, cohort differences (people born in different periods developing different moral priorities), and period effects (historical events or cultural shifts affecting all ages simultaneously). Longitudinal and cohort-sequential

designs that follow multiple birth cohorts across time, as well as repeated cross-sectional surveys collected at multiple time points, would be needed to separate these influences more cleanly.

Second, while self-report measures like the MFQ-2 have established utility, incorporating behavioural measures of moral decision-making and ecologically valid outcomes would provide valuable complementary evidence and help clarify whether the observed differences translate into real-world choices.

Third, our focus on US and UK samples limits generalizability, as cultures can assign different meanings and salience to moral concerns (Atari et al., 2023; Iurino & Saucier, 2018). Future work should examine whether the same age gradients, gender differences, and distributional patterns appear in more diverse cultural contexts.

Finally, the observed floor and ceiling effects—particularly for purity and care—may reflect broader societal influences that deserve systematic investigation, including secularization and religious engagement, socioeconomic change, and educational expansion (Deary et al., 2008; Schoon et al., 2010). One useful direction would be to test whether these distributional patterns shift across historical time using repeated cross-sections or culturally "archaeological" approaches (e.g., tracking moral themes in texts and other cultural artifacts; Baumard, 2018).

Conclusion

In conclusion, in two studies, we found increases in all binding foundations and decreases in support for equality with age, while care scores remained stable across the age range. For gender, women, as predicted, scored higher on both care and equality and lower on three out of four binding foundations (authority, loyalty, proportionality). Contrary to the prediction, no gender differences in purity were found. These findings underscore the importance of considering maturational (Lodi-Smith et al., 2017; Roberts et al., 2008) and societal (Baumard et al., 2024) drivers of moral values.

Data Accessibility Statement

The data and materials used in this paper, as well as the R code used to generate the results, are openly available at the OSF site for this paper at https://osf.io/yk3qc/?view_only=38928cc2387d489cbb22039dc4ecb159

References

Al-Shawaf, L., Lewis, D. M., & Buss, D. M. (2018). Sex differences in disgust: Why are women more easily disgusted than men? *Emotion review*, *10*(2), 149-160.

Amormino, P., Ploe, M. L., & Marsh, A. A. (2022). Moral foundations, values, and judgments in extraordinary altruists. *Scientific Reports*, *12*(1), 22111.

Atari, M., Haidt, J., Graham, J., Koleva, S., Stevens, S. T., & Dehghani, M. (2023). Morality beyond the WEIRD: How the nomological network of morality varies across cultures. *Journal of Personality and Social Psychology*.

Atari, M., Lai, M. H., & Dehghani, M. (2020). Sex differences in moral judgements across 67 countries. *Proceedings of the Royal Society B*, *287*(1937), 20201201.

Bates, T. C., Maes, H., & Neale, M. C. (2019). umx: Twin and Path-Based Structural Equation Modeling in R. *Twin Res Hum Genet*, *22*(1), 27-41. https://doi.org/10.1017/thg.2019.2

Baumard, N. (2018). Psychological origins of the Industrial Revolution. *Behavioral and Brain Sciences*, *42*, e189. https://doi.org/10.1017/S0140525X1800211X

Baumard, N., André, J.-B., & Sperber, D. (2013). A mutualistic approach to morality: The evolution of fairness by partner choice. *Behavioral and brain sciences*, *36*(1), 59-78.

Baumard, N., Safra, L., Martins, M., & Chevallier, C. (2024). Cognitive fossils: using cultural artifacts to reconstruct psychological changes throughout history. *Trends Cogn Sci*, *28*(2), 172-186. https://doi.org/10.1016/j.tics.2023.10.001

Capraro, V. (2020). Gender differences in the trade-off between objective equality and efficiency. *Judgment and Decision Making*, *15*(4), 534-544.

Castilla-Estévez, D., & Blázquez-Rincón, D. (2021). Age and moral foundations: A meta-analytic approach. *The Spanish Journal of Psychology*, *24*, e41.

Clifford, S. (2017). Individual differences in group loyalty predict partisan strength. *Political Behavior*, *39*(3), 531-552.

Costanza, D. P., Rudolph, C. W., & Zacher, H. (2023). Are generations a useful concept? *Acta Psychol (Amst)*, *241*, 104059. https://doi.org/10.1016/j.actpsy.2023.104059

Davies, C. L., Sibley, C. G., & Liu, J. H. (2014). Confirmatory Factor Analysis of the Moral Foundations Questionnaire. *Social Psychology*, *45*(6), 431-436. https://doi.org/10.1027/1864-9335/a000201

Deary, I. J., Batty, G. D., & Gale, C. R. (2008). Bright children become enlightened adults. *Psychol Sci*, *19*(1), 1-6. https://doi.org/10.1111/j.1467-9280.2008.02036.x

Doğruyol, B., Alper, S., & Yilmaz, O. (2019). The five-factor model of the moral foundations theory is stable across WEIRD and non-WEIRD cultures. *Personality and Individual Differences*, *151*. https://doi.org/10.1016/j.paid.2019.109547

Erikson, E. H., Erikson, J. M., & Kivnick, H. Q. (1994). *Vital involvement in old age*. WW Norton & Company.

Fehr, E., Naef, M., & Schmidt, K. M. (2006). Inequality aversion, efficiency, and maximin preferences in simple distribution experiments: Comment. *American Economic Review*, *96*(5), 1912-1917.

Feldman, G. (2021). Personal values and moral foundations: Examining relations and joint prediction of moral variables. *Social Psychological and Personality Science*, *12*(5), 676-686.

Fitouchi, L., André, J.-B., & Baumard, N. (2023). Moral disciplining: The cognitive and evolutionary foundations of puritanical morality. *Behavioral and Brain Sciences*, *46*, e293, Article e293. https://doi.org/10.1017/S0140525X22002047

Freeman, K. J. (1912). *Schools of hellas: an essay on the practice and theory of ancient Greek education from 600 to 300 BC*. Macmillan.

Gao, W., Chen, M., & Li, Y. (2016). Descriptive norms influence reactions to anger-related moral events. *Asian Journal of Social Psychology*, *19*(1), 31-39.

Graham, J., Haidt, J., & Nosek, B. A. (2009). Liberals and conservatives rely on different sets of moral foundations. *J Pers Soc Psychol*, *96*(5), 1029-1046. https://doi.org/10.1037/a0015141

Graham, J., Nosek, B. A., Haidt, J., Iyer, R., Koleva, S., & Ditto, P. H. (2011). Mapping the moral domain. *J Pers Soc Psychol*, *101*(2), 366-385. https://doi.org/10.1037/a0021847

Haidt, J., & Joseph, C. (2004). Intuitive ethics: How innately prepared intuitions generate culturally variable virtues. *Daedalus*, *133*(4), 55-66.

Harnish, R. J., Bridges, K. R., & Gump, J. T. (2018). Predicting economic, social, and foreign policy conservatism: The role of right-wing authoritarianism, social dominance orientation, moral foundations orientation, and religious fundamentalism. *Current Psychology*, *37*, 668-679.

Harper, C. A., & Rhodes, D. (2021). Reanalysing the factor structure of the moral foundations questionnaire. *Br J Soc Psychol*. https://doi.org/10.1111/bjso.12452

Iurino, K., & Saucier, G. (2018). Testing Measurement Invariance of the Moral Foundations Questionnaire Across 27 Countries. *Assessment*, 1073191118817916. https://doi.org/10.1177/1073191118817916

Iyer, R., Koleva, S., Graham, J., Ditto, P., & Haidt, J. (2012). Understanding libertarian morality: the psychological dispositions of self-identified libertarians. *PLoS One*, *7*(8), e42366. https://doi.org/10.1371/journal.pone.0042366

Kivikangas, J. M., Lönnqvist, J.-E., & Ravaja, N. (2017). Relationship of Moral Foundations to Political Liberalism-Conservatism and Left-Right Orientation in a Finnish Representative Sample. *Social Psychology*, *48*(4), 246-251. https://doi.org/10.1027/1864-9335/a000297

Kohlberg, L., & Kramer, R. (1969). Continuities and discontinuities in childhood and adult moral development. *Human Development*, *12*(2), 93-120. https://doi.org/10.1159/000270857

Lin, C.-A., & Bates, T. C. (2022). Free to choose: Mutualist motives for partner choice, proportional division, punishment, and help. *PLoS One*, *17*(5), e0266735.

Lodi-Smith, J., Spain, S. M., Cologgi, K., & Roberts, B. W. (2017). Development of identity clarity and content in adulthood. *Journal of Personality and Social Psychology*, *112*(5), 755.

Milojev, P., Osborne, D., Greaves, L. M., Bulbulia, J., Wilson, M. S., Davies, C. L., Liu, J. H., & Sibley, C. G. (2014). Right-wing authoritarianism and social dominance orientation predict different moral signatures. *Social Justice Research*, *27*, 149-174.

Nilsson, A. (2023). Measurement invariance of moral foundations across population strata. *Journal of Personality Assessment*, *105*(2), 163-173.

Nilsson, A., & Erlandsson, A. (2015). The Moral Foundations taxonomy: Structural validity and relation to political ideology in Sweden. *Personality and Individual Differences*, *76*, 28-32. https://doi.org/10.1016/j.paid.2014.11.049

Nilsson, A., Erlandsson, A., & Västfjäll, D. (2020). Moral foundations theory and the psychology of charitable giving. *European Journal of Personality*, *34*(3), 431-447.

Paruzel-Czachura, M., Pypno, K., & Sorokowski, P. (2023). Alcohol and morality: one alcoholic drink is enough to make people declare to harm others and behave impurely. *Psychopharmacology*, *240*(10), 2163-2172.

Prince, M., Yaprak, A. N., & Palihawadana, D. (2019). The moral bases of consumer ethnocentrism and consumer cosmopolitanism as purchase dispositions. *Journal of Consumer Marketing*, *36*(3), 429-438.

R Core Team. (2023). *R: A language and environment for statistical computing*. In R Foundation for Statistical Computing, Vienna, Austria. https://www.R-project.org/

Roberts, B. W., Wood, D., & Caspi, A. (2008). The development of personality traits in adulthood.

Schoon, I., Cheng, H., Gale, C. R., Batty, G. D., & Deary, I. J. (2010). Social status, cognitive ability, and educational attainment as predictors of liberal social attitudes and political trust. *Intelligence*, *38*(1), 144-150.

Selterman, D., & Koleva, S. (2015). Moral judgment of close relationship behaviors. *Journal of Social and Personal Relationships*, *32*(7), 922-945.

Trivers, R. L. (1971). The Evolution of Reciprocal Altruism. *The Quarterly Review of Biology*, *46*(1), 35-57. https://doi.org/10.1086/406755

Tybur, J. M., Lieberman, D., & Griskevicius, V. (2009). Microbes, mating, and morality: individual differences in three functional domains of disgust. *Journal of Personality and Social Psychology*, *97*(1), 103.

Van Leeuwen, F., Dukes, A., Tybur, J. M., & Park, J. H. (2017). Disgust sensitivity relates to moral foundations independent of political ideology. *Evolutionary Behavioral Sciences*, *11*(1), 92.

Van Tongeren, D. R., DeWall, C. N., Hardy, S. A., & Schwadel, P. (2021). Religious identity and morality: Evidence for religious residue and decay in moral foundations. *Personality and Social Psychology Bulletin*, *47*(11), 1550-1564.

Vecina, M. L., & Piñuela, R. (2017). Relationships between ambivalent sexism and the five moral foundations in domestic violence: Is it a matter of fairness and authority? *The Journal of psychology*, *151*(3), 334-344.

Wolff, J. (1998). Fairness, respect, and the egalitarian ethos. *Philosophy & public affairs*, *27*(2), 97-122.

Yalçındağ, B., Özkan, T., Cesur, S., Yilmaz, O., Tepe, B., Piyale, Z. E., Biten, A. F., & Sunar, D. (2017). An Investigation of Moral Foundations Theory in Turkey Using Different Measures. *Current Psychology*, *38*(2), 440-457. https://doi.org/10.1007/s12144-017-9618-4

Zakharin, M., & Bates, T. C. (2021). Remapping the foundations of morality: Well-fitting structural model of the Moral Foundations Questionnaire. *PLoS One*, *16*(10), e0258910. https://doi.org/10.1371/journal.pone.0258910

Zakharin, M., & Bates, T. C. (2023). Moral Foundations Theory: Validation and replication of the MFQ-2. *Personality and Individual Differences*, *214*, 112339.

Zakharin, M., & Bates, T. C. (2024a). Bright people have weaker moral foundations [Manuscript submitted for publication]. *Intelligence*.

Zakharin, M., & Bates, T. C. (2024b). Psychological pillars of support for free speech: Tolerance for offensive, disagreeing, socially divisive, and heterodox speech. *Personality and Individual Differences*, *219*. https://doi.org/10.1016/j.paid.2023.112502

Cover Letter / Response to Reviewers

Manuscript ID: PONE-D-24-60347

Title: Age, gender, and distributional effects on moral foundations

Authors: Michael Zakharin & Timothy C. Bates

Dear Dr. Carrasco-Farré,

Thank you for the opportunity to revise our manuscript. We are grateful to you and the reviewers for the positive evaluation of our design and methodology, and for the constructive feedback regarding the theoretical framing and discussion of implications.

We have addressed all points raised. Specifically, we have:

1. Clarified the theoretical basis for our hypotheses regarding proportionality and effect sizes.
2. Expanded the discussion on the societal and political implications of these age/gender gaps.
3. Elaborated on the "maturational vs. societal" mechanisms in the Conclusion.
4. Provided detailed definitions of the six MFQ-2 domains and clarified the "distributional effects" terminology throughout the manuscript.
5. Added explicit text regarding cross-sectional design limitations.

Below is a detailed point-by-point response to the Academic Editor and Reviewers.

Response to the Academic Editor

Editor Point 1: Clarify and motivate the hypotheses more explicitly.

"As Reviewer 1 notes, it would be helpful to expand on the reasoning behind specific predictions, such as why proportionality was expected to show a male advantage, and why you anticipated age-related increases across all foundations with effect sizes in the .05–.25 range."

Response:

We agree that the specific predictions for Proportionality (a new foundation in MFQ-2) and the specific effect size estimates required stronger grounding in the introduction.

- **Regarding Proportionality:** We have added text explaining that proportionality aligns with "equity" or meritocratic allocation, a domain where men consistently score higher in evolutionary psychology and economic game literature, contrasting with the "equality" domain where women typically score higher.

- **Regarding Effect Sizes:** We have clarified that the range estimate (.05–.25) was derived directly from the meta-analytic baselines established by Castilla-Estévez & Blázquez-Rincón (2021) and the specific findings of Milojev et al. (2014).

Change to Manuscript:

Original text: " Drawing on these MFQ-2 findings and the broader MFQ-1 literature, we hypothesized..."

New text: "Drawing on MFQ-2 findings and the broader MFQ-1 literature, we hypothesized that our study would show age-related increases across all foundations (effect sizes ranging from .05 to .25), except possibly equality, consistent with the distribution of age effects reported in large-scale MFQ-1 studies (Castilla-Estévez & Blázquez-Rincón, 2021). Given that proportionality is part of the binding domain in MFQ-2, we expected its age association to be comparable in magnitude to the other binding foundations. Regarding gender, we predicted women would score higher on the individualizing foundations (care and equality) and purity, while men would show higher scores on the binding foundations (proportionality, loyalty, and authority), consistent with prior MFQ-1 results and with Atari et al. (2023) who reported a modest male advantage in MFQ-2 proportionality. We further expected a male advantage in proportionality because it aligns more closely with equality- or merit-based allocation; across economic-game paradigms, men tend to endorse merit more and women equality more (Capraro, 2020; Fehr et al., 2006)." (page 6, lines 119 - 130)

Editor Point 2: Elaborate the implications of the observed age and gender differences.

"Reviewer 1 also asks for more discussion of how these differences in moral foundations may translate into broader social and political outcomes... Deepening this part of the Discussion will help readers see the societal relevance of your findings."

Response:

We have expanded the Discussion to explicitly link these psychometric differences to real-world phenomena, specifically political polarization and intergenerational value conflict (e.g., "culture wars").

Change to Manuscript:

New text: "These divergences have tangible societal implications. The sharp age-related decline in Equality concerns, combined with the rise in Binding foundations, suggests a mechanism for the well-documented conservative shift associated with aging. Furthermore, the 0.78 SD gap in binding scores between the youngest and oldest cohorts provides a quantitative basis for current intergenerational conflicts ('culture wars'). Where younger cohorts may view the prioritization of tradition and authority as inherently oppressive, older cohorts may view the dismissal of these

foundations as a failure of moral duty. Such foundation-level disconnects complicate cross-generational political consensus." (page 12, lines 251 - 258)

Editor Point 3: Develop the point about maturational versus societal causes.

"Please expand this argument: outline what kinds of maturational mechanisms you have in mind, what societal changes might be relevant, and whether you would expect these to operate similarly or differently in the US and UK contexts."

Response:

We have expanded the Conclusion to distinguish between Social Investment Theory (maturation) and secular liberalization (societal change). We also noted that given the shared "WEIRD" (Western, Educated, Industrialized, Rich, Democratic) status of the US and UK, we expect similar mechanisms in both, though the baseline levels might differ.

Change to Manuscript (Conclusion):

Original text: "Findings underscore the importance of considering both maturational... and societal... causes as drivers of changes in moral values."

New text: "These findings underscore the importance of considering both maturational and societal drivers. Maturational mechanisms likely include 'social investment' processes, where taking on adult roles (parenthood, employment) necessitates increased valuation of stability, authority, and loyalty. Simultaneously, societal drivers likely reflect the 'Flynn effect for morality' or broad secular liberalization, pushing cohort means toward higher Care and lower Purity. Given their shared cultural and economic structures, we expect these dual pressures to operate similarly across US and UK contexts, though their relative strength may vary with national factors such as religiosity and political culture." (page 13, lines 275 - 282)

Editor Point 4: Provide more background on the MFQ-1/MFQ-2 and the moral domains.

"Please elaborate briefly on the six moral domains... and clarify how MFQ-2 differs from MFQ-1... provide the MFQ-1 and MFQ-2 item sets as Supporting Information files..."

Response:

We have expanded the introduction to clearly define the six domains. We have also uploaded the full item sets for MFQ-1 and MFQ-2 as a Supporting Information file (S1_File_MFQ_Items.pdf and S2_File_MFQ -2_Items.pdf) as requested.

Change to Manuscript:

Original text: "The MFQ-2 differs from the MFQ-1 in two notable ways…"

New text: " Moral foundations theory (MFT; Atari et al., 2023; Graham et al., 2011; Haidt & Joseph, 2004) proposes that moral judgment is supported by multiple partially distinct concerns rather than a single moral principle. In this paper we focus on the six foundations assessed by the MFQ-2: care, equality, proportionality, loyalty, authority, and purity. Care captures concern for others' suffering and harm prevention. Equality reflects egalitarian concern that people should be treated similarly and have similar outcomes. Proportionality reflects merit-based fairness - benefits and punishments should track effort, contribution, or responsibility (conceptually related to mutualism; Baumard et al., 2013; Lin & Bates, 2022). Loyalty concerns commitment to one's group and aversion to betrayal. Authority concerns respect for legitimate hierarchy, tradition, and role-based duties. Purity concerns contamination and sanctity, including bodily and spiritual cleanliness and self-control. These foundations are often summarized into higher-order clusters in Western samples, with individualizing concerns (care, equality) emphasizing protection and equal regard for individuals, and binding concerns (loyalty, authority, purity, and proportionality) emphasizing group cohesion, order, and norm enforcement. (Atari et al., 2023; Graham et al., 2011).

Moral foundations are commonly measured with the MFQ-1 (Graham et al., 2011) and the updated MFQ-2 (Atari et al., 2023). The key conceptual difference is that MFQ-1 assesses five foundations, including a single fairness scale, whereas recently developed MFQ-2 assesses six foundations by splitting fairness into two separable constructs: equality and proportionality. In addition, MFQ-2 replaces the original MFQ-1 items with newly written or rephrased items, intended to improve clarity and content coverage and to better recover the expected factor structure in contemporary samples (Atari et al., 2023; Zakharin & Bates, 2023), addressing well-documented psychometric limitations of MFQ-1 in some datasets (e.g. Davies et al., 2014; Doğruyol et al., 2019; Iurino & Saucier, 2018; Kivikangas et al., 2017).The complete item sets for MFQ-1 and MFQ-2 are provided in the Supporting Information." (page 4, lines 67 - 74)

Editor Point 5: Clarify the "distributional effects" framing.

"The title and framing emphasise "distributional effects," but the meaning of this term... could be described more explicitly."

Response:

We used "distributional effects" to refer specifically to the shape of the score distributions (skewness/kurtosis) and the identification of ceiling/floor effects, rather than variance heterogeneity. We have clarified this definition in the Introduction and Results to avoid ambiguity. We also changed the title to "Age, gender, and score distributions of moral foundations"

Change to Manuscript:

New text (Introduction): "... to describe mean differences in moral foundations by age and gender and the shape of MFQ-2 scores in the population—specifically examining

distributional properties such as skew and ceiling/floor effects—and the changes exhibited with age." (page 3, lines 49 - 52)

New text (Results): "For each foundation (and the binding/individualizing composites), we summarized score distributions using the mean and SD and quantified distributional shape using skewness and kurtosis. We also inspected histograms to identify clustering near the minimum or maximum response options (floor/ceiling effects)" (page 9, lines 167 - 171)

Editor Point 6: Multiple comparisons and effect size interpretation.

"State how you addressed the issue of multiple testing... consider adding a short paragraph in the Discussion that contextualises the magnitude of the observed effects"

Response:

Given the large sample size (N > 2400 total) and the exploratory nature of the demographic mapping, we prioritized estimation of effect sizes (beta) over strict p-value thresholding, though we note that our key findings are significant at $p < .001$, robust to Bonferroni correction for the 12 primary tests (6 domains × 2 demographic variables).

Change to Manuscript (Results):

New text: "Given the number of comparisons across six moral foundations, two predictors (age, gender), and two samples, we prioritized the magnitude and consistency of standardized coefficients (β) over reliance on individual p-values. Nonetheless, we note that the majority of primary effects reported below survive a Bonferroni-corrected threshold of $\alpha = .05/12 = .004$ per sample, and effects that replicated across both samples provide stronger evidence than any single test." (page 10, lines 204 - 209)

Editor Point 7: Age, cohort, and design limitations.

"Because the data are cross-sectional, age effects are potentially confounded with cohort and period effects... acknowledge this explicitly in the Limitations section"

Response:

We have reinforced the Limitations section to explicitly state the Age/Period/Cohort confound.

Change to Manuscript:

Original text: "Our cross-sectional design cannot disentangle age effects from cohort effects..."

New text: "The associations we report are between-person age differences and cannot be taken as evidence of within-person developmental (maturational) change. Age is confounded with birth cohort and historical period: older participants differ not only in age but also in the social and cultural context in which they were raised. Thus, the observed age-related increase in binding foundations (and decrease in equality) could reflect maturation, cohort-based socialization, or both. Longitudinal—ideally cohort-sequential—data are needed to separate age, cohort, and period influences." (page 13, lines 291 - 297)

Response to Reviewers

Reviewer #1

We thank Reviewer 1 for their assessment that the manuscript is "well-written and well-designed"[15].

R1 Point 1: Explain hypotheses (Proportionality male advantage, effect sizes). 16

Response: As detailed in our response to the Editor (Point 1), we have revised the Introduction to explicitly ground the Proportionality hypothesis in literature suggesting male preferences for equity/meritocracy over equality and justified the effect size prediction based on the Castilla-Estévez meta-analysis.

R1 Point 2: Discuss repercussions/implications.

Response: We have expanded the Discussion to address political affiliations and intergenerational value conflict, as requested. (See response to Editor Point 2).

R1 Point 3: Expand on maturational vs. societal causes.

Response: We have elaborated on the Conclusion to contrast social investment mechanisms against secular shifts in values. (See response to Editor Point 3).

Reviewer #2

We thank Reviewer 2 for their comments regarding the solid analysis.

R2 Point 1: Provide a copy of the MFQ-1 and MFQ-2. 20

Response: We have created a Supporting Information files containing the items for the MFQ-1 and MFQ-2 to assist readers in understanding the instrument.

R2 Point 2: Elaborate on the different domains.

Response: We have revised the Introduction to provide distinct, conceptual definitions for Care, Equality, Proportionality, Loyalty, Authority, and Purity. (See response to Editor Point 4).

Specific Ethics Statement Update

Per Journal Requirement #222, we have updated the Methods section:

" The present study is a secondary analysis of previously collected data that are publicly available in de-identified form. The original data collection was approved by the Psychology Research Ethics Committee at the University of Edinburgh. Informed consent was obtained from all participants electronically prior to data collection. The current analyses used only anonymized, publicly shared data and therefore did not involve additional participant contact or collection of identifiable information."

Data Availability

Data remains available as per the original submission.

We believe these revisions significantly strengthen the manuscript and look forward to your decision.

Sincerely,

Michael Zakharin & Timothy C. Bates

Department of Psychology, University of Edinburgh
