# Supplementary material for: Age, gender, and score distributions of moral foundations
Source: PLoS One. 2026 Jul 1;21(7):e0352584. doi: 10.1371/journal.pone.0352584 (PMC13322558; doi:10.1371/journal.pone.0352584)
Supplement: Attachment — Submitted filename: 2 Response to reviewers.docx [file pone.0352584.s003.docx]

**Cover Letter / Response to Reviewers**

Manuscript ID: PONE-D-24-60347

Title: Age, gender, and distributional effects on moral foundations

Authors: Michael Zakharin & Timothy C. Bates

**Dear Dr. Carrasco-Farré,**

Thank you for the opportunity to revise our manuscript. We are grateful to you and the reviewers for the positive evaluation of our design and methodology, and for the constructive feedback regarding the theoretical framing and discussion of implications.

We have addressed all points raised. Specifically, we have:

1. Clarified the theoretical basis for our hypotheses regarding proportionality and effect sizes.
2. Expanded the discussion on the societal and political implications of these age/gender gaps.
3. Elaborated on the "maturational vs. societal" mechanisms in the Conclusion.
4. Provided detailed definitions of the six MFQ-2 domains and clarified the "distributional effects" terminology throughout the manuscript.
5. Added explicit text regarding cross-sectional design limitations.

Below is a detailed point-by-point response to the Academic Editor and Reviewers.

**Response to the Academic Editor**

**Editor Point 1: Clarify and motivate the hypotheses more explicitly.**

*"As Reviewer 1 notes, it would be helpful to expand on the reasoning behind specific predictions, such as why proportionality was expected to show a male advantage, and why you anticipated age-related increases across all foundations with effect sizes in the .05–.25 range."*

Response:

We agree that the specific predictions for Proportionality (a new foundation in MFQ-2) and the specific effect size estimates required stronger grounding in the introduction.

- **Regarding Proportionality:** We have added text explaining that proportionality aligns with "equity" or meritocratic allocation, a domain where men consistently score higher in evolutionary psychology and economic game literature, contrasting with the "equality" domain where women typically score higher.
- **Regarding Effect Sizes:** We have clarified that the range estimate (.05–.25) was derived directly from the meta-analytic baselines established by Castilla-Estévez & Blázquez-Rincón (2021) and the specific findings of Milojev et al. (2014).

Change to Manuscript:

Original text: " Drawing on these MFQ-2 findings and the broader MFQ-1 literature, we hypothesized..."

New text: "Drawing on MFQ-2 findings and the broader MFQ-1 literature, we hypothesized that our study would show age-related increases across all foundations (effect sizes ranging from .05 to .25), except possibly equality, consistent with the distribution of age effects reported in large-scale MFQ-1 studies (Castilla-Estévez & Blázquez-Rincón, 2021). Given that proportionality is part of the binding domain in MFQ-2, we expected its age association to be comparable in magnitude to the other binding foundations. Regarding gender, we predicted women would score higher on the individualizing foundations (care and equality) and purity, while men would show higher scores on the binding foundations (proportionality, loyalty, and authority), consistent with prior MFQ-1 results and with Atari et al. (2023) who reported a modest male advantage in MFQ-2 proportionality. We further expected a male advantage in proportionality because it aligns more closely with equality- or merit-based allocation; across economic-game paradigms, men tend to endorse merit more and women equality more (Capraro, 2020; Fehr et al., 2006)." (page 6, lines 119 - 130)

**Editor Point 2: Elaborate the implications of the observed age and gender differences.**

*"Reviewer 1 also asks for more discussion of how these differences in moral foundations may translate into broader social and political outcomes... Deepening this part of the Discussion will help readers see the societal relevance of your findings."*

Response:

We have expanded the Discussion to explicitly link these psychometric differences to real-world phenomena, specifically political polarization and intergenerational value conflict (e.g., "culture wars").

Change to Manuscript:

New text: "These divergences have tangible societal implications. The sharp age-related decline in Equality concerns, combined with the rise in Binding foundations, suggests a mechanism for the well-documented conservative shift associated with aging. Furthermore, the 0.78 SD gap in binding scores between the youngest and oldest cohorts provides a quantitative basis for current intergenerational conflicts ('culture wars'). Where younger cohorts may view the prioritization of tradition and authority as inherently oppressive, older cohorts may view the dismissal of these foundations as a failure of moral duty. Such foundation-level disconnects complicate cross-generational political consensus." (page 12, lines 251 - 258)

**Editor Point 3: Develop the point about maturational versus societal causes.**

*"Please expand this argument: outline what kinds of maturational mechanisms you have in mind, what societal changes might be relevant, and whether you would expect these to operate similarly or differently in the US and UK contexts."*

Response:

We have expanded the Conclusion to distinguish between Social Investment Theory (maturation) and secular liberalization (societal change). We also noted that given the shared "WEIRD" (Western, Educated, Industrialized, Rich, Democratic) status of the US and UK, we expect similar mechanisms in both, though the baseline levels might differ.

Change to Manuscript (Conclusion):

Original text: "Findings underscore the importance of considering both maturational... and societal... causes as drivers of changes in moral values."

New text: "These findings underscore the importance of considering both maturational and societal drivers. Maturational mechanisms likely include 'social investment' processes, where taking on adult roles (parenthood, employment) necessitates increased valuation of stability, authority, and loyalty. Simultaneously, societal drivers likely reflect the 'Flynn effect for morality' or broad secular liberalization, pushing cohort means toward higher Care and lower Purity. Given their shared cultural and economic structures, we expect these dual pressures to operate similarly across US and UK contexts, though their relative strength may vary with national factors such as religiosity and political culture." (page 13, lines 275 - 282)

**Editor Point 4: Provide more background on the MFQ-1/MFQ-2 and the moral domains.**

*"Please elaborate briefly on the six moral domains... and clarify how MFQ-2 differs from MFQ-1... provide the MFQ-1 and MFQ-2 item sets as Supporting Information files..."*

Response:

We have expanded the introduction to clearly define the six domains. We have also uploaded the full item sets for MFQ-1 and MFQ-2 as a Supporting Information file (S1_File_MFQ_Items.pdf and S2_File_MFQ -2_Items.pdf) as requested.

Change to Manuscript:

Original text: "The MFQ-2 differs from the MFQ-1 in two notable ways…"

New text: " Moral foundations theory (MFT; Atari et al., 2023; Graham et al., 2011; Haidt & Joseph, 2004) proposes that moral judgment is supported by multiple partially distinct concerns rather than a single moral principle. In this paper we focus on the six foundations assessed by the MFQ-2: care, equality, proportionality, loyalty, authority, and purity. Care captures concern for others’ suffering and harm prevention. Equality reflects egalitarian concern that people should be treated similarly and have similar outcomes. Proportionality reflects merit-based fairness - benefits and punishments should track effort, contribution, or responsibility (conceptually related to mutualism; Baumard et al., 2013; Lin & Bates, 2022). Loyalty concerns commitment to one’s group and aversion to betrayal. Authority concerns respect for legitimate hierarchy, tradition, and role-based duties. Purity concerns contamination and sanctity, including bodily and spiritual cleanliness and self-control. These foundations are often summarized into higher-order clusters in Western samples, with individualizing concerns (care, equality) emphasizing protection and equal regard for individuals, and binding concerns (loyalty, authority, purity, and proportionality) emphasizing group cohesion, order, and norm enforcement. (Atari et al., 2023; Graham et al., 2011).

Moral foundations are commonly measured with the MFQ-1 (Graham et al., 2011) and the updated MFQ-2 (Atari et al., 2023). The key conceptual difference is that MFQ-1 assesses five foundations, including a single fairness scale, whereas recently developed MFQ-2 assesses six foundations by splitting fairness into two separable constructs: equality and proportionality. In addition, MFQ-2 replaces the original MFQ-1 items with newly written or rephrased items, intended to improve clarity and content coverage and to better recover the expected factor structure in contemporary samples (Atari et al., 2023; Zakharin & Bates, 2023), addressing well-documented psychometric limitations of MFQ-1 in some datasets (e.g. Davies et al., 2014; Doğruyol et al., 2019; Iurino & Saucier, 2018; Kivikangas et al., 2017).The complete item sets for MFQ-1 and MFQ-2 are provided in the Supporting Information." (page 4, lines 67 - 74)

**Editor Point 5: Clarify the “distributional effects” framing.**

*"The title and framing emphasise “distributional effects,” but the meaning of this term... could be described more explicitly."*

Response:

We used "distributional effects" to refer specifically to the shape of the score distributions (skewness/kurtosis) and the identification of ceiling/floor effects, rather than variance heterogeneity. We have clarified this definition in the Introduction and Results to avoid ambiguity. We also changed the title to “Age, gender, and score distributions of moral foundations”

Change to Manuscript:

New text (Introduction): "... to describe mean differences in moral foundations by age and gender and the shape of MFQ-2 scores in the population—specifically examining distributional properties such as skew and ceiling/floor effects—and the changes exhibited with age." (page 3, lines 49 - 52)

New text (Results): "For each foundation (and the binding/individualizing composites), we summarized score distributions using the mean and SD and quantified distributional shape using skewness and kurtosis. We also inspected histograms to identify clustering near the minimum or maximum response options (floor/ceiling effects)" (page 9, lines 167 - 171)

**Editor Point 6: Multiple comparisons and effect size interpretation.**

*"State how you addressed the issue of multiple testing... consider adding a short paragraph in the Discussion that contextualises the magnitude of the observed effects"*

Response:

Given the large sample size (N > 2400 total) and the exploratory nature of the demographic mapping, we prioritized estimation of effect sizes (beta) over strict p-value thresholding, though we note that our key findings are significant at p < .001, robust to Bonferroni correction for the 12 primary tests (6 domains × 2 demographic variables).

Change to Manuscript (Results):

New text: "Given the number of comparisons across six moral foundations, two predictors (age, gender), and two samples, we prioritized the magnitude and consistency of standardized coefficients (β) over reliance on individual p-values. Nonetheless, we note that the majority of primary effects reported below survive a Bonferroni-corrected threshold of α = .05/12 = .004 per sample, and effects that replicated across both samples provide stronger evidence than any single test." (page 10, lines 204 - 209)

**Editor Point 7: Age, cohort, and design limitations.**

*"Because the data are cross-sectional, age effects are potentially confounded with cohort and period effects... acknowledge this explicitly in the Limitations section"*

Response:

We have reinforced the Limitations section to explicitly state the Age/Period/Cohort confound.

Change to Manuscript:

Original text: "Our cross-sectional design cannot disentangle age effects from cohort effects..."

New text: "The associations we report are between-person age differences and cannot be taken as evidence of within-person developmental (maturational) change. Age is confounded with birth cohort and historical period: older participants differ not only in age but also in the social and cultural context in which they were raised. Thus, the observed age-related increase in binding foundations (and decrease in equality) could reflect maturation, cohort-based socialization, or both. Longitudinal—ideally cohort-sequential—data are needed to separate age, cohort, and period influences." (page 13, lines 291 - 297)

**Response to Reviewers**

**Reviewer #1**

We thank Reviewer 1 for their assessment that the manuscript is "well-written and well-designed"^15^.

R1 Point 1: Explain hypotheses (Proportionality male advantage, effect sizes). 16

Response: As detailed in our response to the Editor (Point 1), we have revised the Introduction to explicitly ground the Proportionality hypothesis in literature suggesting male preferences for equity/meritocracy over equality and justified the effect size prediction based on the Castilla-Estévez meta-analysis.

R1 Point 2: Discuss repercussions/implications.

Response: We have expanded the Discussion to address political affiliations and intergenerational value conflict, as requested. (See response to Editor Point 2).

R1 Point 3: Expand on maturational vs. societal causes.

Response: We have elaborated on the Conclusion to contrast social investment mechanisms against secular shifts in values. (See response to Editor Point 3).

**Reviewer #2**

*We thank Reviewer 2 for their comments regarding the solid analysis.*

R2 Point 1: Provide a copy of the MFQ-1 and MFQ-2. 20

Response: We have created a Supporting Information files containing the items for the MFQ-1 and MFQ-2 to assist readers in understanding the instrument.

R2 Point 2: Elaborate on the different domains.

Response: We have revised the Introduction to provide distinct, conceptual definitions for Care, Equality, Proportionality, Loyalty, Authority, and Purity. (See response to Editor Point 4).

Specific Ethics Statement Update

Per Journal Requirement #222, we have updated the Methods section:

" The present study is a secondary analysis of previously collected data that are publicly available in de-identified form. The original data collection was approved by the Psychology Research Ethics Committee at the University of Edinburgh. Informed consent was obtained from all participants electronically prior to data collection. The current analyses used only anonymized, publicly shared data and therefore did not involve additional participant contact or collection of identifiable information."

Data Availability

Data remains available as per the original submission.

We believe these revisions significantly strengthen the manuscript and look forward to your decision.

Sincerely,

Michael Zakharin & Timothy C. Bates

Department of Psychology, University of Edinburgh
