# Supplementary material for: Age, gender, and score distributions of moral foundations
Source: PLoS One. 2026 Jul 1;21(7):e0352584. doi: 10.1371/journal.pone.0352584 (PMC13322558; doi:10.1371/journal.pone.0352584)
Supplement: Peer review history

# Peer review: Decision Letter 0

**DOI:** 10.1371/journal.pone.0352584.r001

**Authors:** Carlos Carrasco-Farré

29 Nov 2025

PONE-D-24-60347

Age, gender, and distributional effects on moral foundations

PLOS ONE

Dear Dr. Zakharin,

Thank you for submitting your manuscript “Age, gender, and distributional effects on moral foundations” to PLOS ONE. We have now received comments from two expert reviewers, and I have considered these alongside my own reading of the paper.

Both reviewers are positive about the quality of the work, describing the manuscript as well-designed, clearly written, and methodologically sound. Their suggestions focus on clarifying and expanding the theoretical background and discussion, as well as providing additional background on the measures, rather than on changes to your design or analyses.

On this basis, I am pleased to invite you to submit a revised manuscript, and I am designating the decision as Minor Revision.

In preparing your revision, please address the reviewers’ points in a detailed, point-by-point response. In particular, I encourage you to:

1. Clarify and motivate the hypotheses more explicitly.

As Reviewer 1 notes, it would be helpful to expand on the reasoning behind specific predictions, such as why proportionality was expected to show a male advantage, and why you anticipated age-related increases across all foundations with effect sizes in the .05–.25 range. Connecting these expectations more clearly to prior empirical work or theoretical arguments would strengthen the Introduction.

2. Elaborate the implications of the observed age and gender differences.

Reviewer 1 also asks for more discussion of how these differences in moral foundations may translate into broader social and political outcomes (e.g., patterns of political affiliation, intergenerational value conflict). Deepening this part of the Discussion will help readers see the societal relevance of your findings.

3. Develop the point about maturational versus societal causes.

In the Conclusion you note that your results underscore the importance of both maturational and societal drivers of moral change. Please expand this argument: outline what kinds of maturational mechanisms you have in mind, what societal changes might be relevant, and whether you would expect these to operate similarly or differently in the US and UK contexts.

4. Provide more background on the MFQ-1/MFQ-2 and the moral domains.

In line with Reviewer 2’s comments, please elaborate briefly on the six moral domains (care, equality/fairness, proportionality, loyalty, authority, purity) and clarify how MFQ-2 differs from MFQ-1.

It would be very useful to provide the MFQ-1 and MFQ-2 item sets as Supporting Information files, or at minimum to include a clear description that allows readers unfamiliar with these instruments to fully understand the differences.

In addition to the reviewers’ points, I would also ask you to consider the following issues raised from my side as Academic Editor:

5. Clarify the “distributional effects” framing.

The title and framing emphasise “distributional effects,” but the meaning of this term (e.g., heterogeneity across age groups, variation across the distribution of scores, or differences in variance) could be described more explicitly. Please ensure that the Introduction, Methods, and Results clearly explain what you mean by distributional effects and how your analyses capture them.

6. Multiple comparisons and effect size interpretation.

Given the number of moral foundations and demographic comparisons, it would be helpful to briefly state how you addressed the issue of multiple testing (e.g., correction procedures, emphasis on effect sizes rather than single p-values).

Relatedly, please consider adding a short paragraph in the Discussion that contextualises the magnitude of the observed effects (e.g., small but consistent differences) and avoids any possible overinterpretation.

7. Age, cohort, and design limitations.

Because the data are cross-sectional, age effects are potentially confounded with cohort and period effects. It would strengthen the manuscript to acknowledge this explicitly in the Limitations section and to clarify what kinds of inferences can and cannot be drawn about developmental (maturational) change versus cohort differences.

Please submit your revised manuscript by Jan 13 2026 11:59PM. If you will need more time than this to complete your revisions, please reply to this message or contact the journal office at plosone@plos.org. When you're ready to submit your revision, log on to https://www.editorialmanager.com/pone/ and select the 'Submissions Needing Revision' folder to locate your manuscript file.

Please include the following items when submitting your revised manuscript:

- A rebuttal letter that responds to each point raised by the academic editor and reviewer(s). You should upload this letter as a separate file labeled 'Response to Reviewers'.
- A marked-up copy of your manuscript that highlights changes made to the original version. You should upload this as a separate file labeled 'Revised Manuscript with Track Changes'.
- An unmarked version of your revised paper without tracked changes. You should upload this as a separate file labeled 'Manuscript'.

-->If you would like to make changes to your financial disclosure, please include your updated statement in your cover letter. Guidelines for resubmitting your figure files are available below the reviewer comments at the end of this letter.

If applicable, we recommend that you deposit your laboratory protocols in protocols.io to enhance the reproducibility of your results. Protocols.io assigns your protocol its own identifier (DOI) so that it can be cited independently in the future. For instructions see: https://journals.plos.org/plosone/s/submission-guidelines#loc-laboratory-protocols. Additionally, PLOS ONE offers an option for publishing peer-reviewed Lab Protocol articles, which describe protocols hosted on protocols.io. Read more information on sharing protocols at https://plos.org/protocols?utm_medium=editorial-email&utm_source=authorletters&utm_campaign=protocols.

We look forward to receiving your revised manuscript.

Kind regards,

Carlos Carrasco-Farré

Academic Editor

PLOS ONE

Journal Requirements:

When submitting your revision, we need you to address these additional requirements.

1. Please ensure that your manuscript meets PLOS ONE's style requirements, including those for file naming. The PLOS ONE style templates can be found at

https://journals.plos.org/plosone/s/file?id=wjVg/PLOSOne_formatting_sample_main_body.pdf and

https://journals.plos.org/plosone/s/file?id=ba62/PLOSOne_formatting_sample_title_authors_affiliations.pdf

2. Please include your full ethics statement in the ‘Methods’ section of your manuscript file. In your statement, please include the full name of the IRB or ethics committee who approved or waived your study, as well as whether or not you obtained informed written or verbal consent. If consent was waived for your study, please include this information in your statement as well.

3. We noted in your submission details that a portion of your manuscript may have been presented or published elsewhere. Please clarify whether this publication was peer-reviewed and formally published. If this work was previously peer-reviewed and published, in the cover letter please provide the reason that this work does not constitute dual publication and should be included in the current manuscript.

4. If the reviewer comments include a recommendation to cite specific previously published works, please review and evaluate these publications to determine whether they are relevant and should be cited. There is no requirement to cite these works unless the editor has indicated otherwise.

5. Please review your reference list to ensure that it is complete and correct. If you have cited papers that have been retracted, please include the rationale for doing so in the manuscript text, or remove these references and replace them with relevant current references. Any changes to the reference list should be mentioned in the rebuttal letter that accompanies your revised manuscript. If you need to cite a retracted article, indicate the article’s retracted status in the References list and also include a citation and full reference for the retraction notice.

[Note: HTML markup is below. Please do not edit.]

Reviewers' comments:

Reviewer's Responses to Questions

**Comments to the Author**

1. Is the manuscript technically sound, and do the data support the conclusions?

The manuscript must describe a technically sound piece of scientific research with data that supports the conclusions. Experiments must have been conducted rigorously, with appropriate controls, replication, and sample sizes. The conclusions must be drawn appropriately based on the data presented.

Reviewer #1: Yes

Reviewer #2: Yes

**********

2. Has the statistical analysis been performed appropriately and rigorously?

Reviewer #1: Yes

Reviewer #2: Yes

**********

3. Have the authors made all data underlying the findings in their manuscript fully available?

The PLOS Data policy requires authors to make all data underlying the findings described in their manuscript fully available without restriction, with rare exception (please refer to the Data Availability Statement in the manuscript PDF file). The data should be provided as part of the manuscript or its supporting information, or deposited to a public repository. For example, in addition to summary statistics, the data points behind means, medians and variance measures should be available. If there are restrictions on publicly sharing data—e.g. participant privacy or use of data from a third party—those must be specified.

Reviewer #1: Yes

Reviewer #2: Yes

**********

4. Is the manuscript presented in an intelligible fashion and written in standard English?

PLOS ONE does not copyedit accepted manuscripts, so the language in submitted articles must be clear, correct, and unambiguous. Any typographical or grammatical errors should be corrected at revision, so please note any specific errors here.

Reviewer #1: Yes

Reviewer #2: Yes

**********

5. Review Comments to the Author

Please use the space provided to explain your answers to the questions above. You may also include additional comments for the author, including concerns about dual publication, research ethics, or publication ethics. (Please upload your review as an attachment if it exceeds 20,000 characters)

Reviewer #1: The present manuscript, “Age, gender, and distributional effects on moral foundations,” reports two studies examining the effects of age and gender on six moral concerns: Care, Equality, Proportionality, Loyalty, Authority, and Purity. The authors rely on correlations drawn from the Moral Foundations Questionnaire-2 (MFQ-2), showing increasing and decreasing trends amongst women and men (both young and old) to capture changes in moral values. I enjoyed reading this manuscript; the Introduction is concise, the Methods are robust, and the Results are well-explained. The paper is well-written and well-designed overall. In what follows, I have listed a number of minor points and suggestions, which I hope the authors will find helpful in revising their work.

1. Although great so far, the authors would benefit from further explaining their hypotheses. For example, why did the authors predict that “proportionality would show a male advantage,” or why did they anticipate age-related increases across all moral foundations with “effect sizes ranging from .05 to .25”? Seems a bit arbitrary without knowing their thought process. Elaborating a bit would help clear the air.

2. Moreover, it would be helpful (and interesting) if the authors discuss and cite work that considers the repercussions of the age and gender differences found amongst the moral foundations. I concur with their saying of how these differences “result in substantially different moral worldviews between younger and older adults.” However, it would be great if they dug a little deeper and expanded. How can this help us understand, for instance, the political affiliations these different groups are drawn to?

3. Lastly, in the authors’ conclusion, they state that these findings are of paramount importance when considering “both maturational and societal causes as drivers of changes in moral values.” This is an interesting point that, again, I believe warrants more explanation. What could these maturational and societal causes be? Would they be the same in the US and the UK?

Reviewer #2: Overall the manuscript provides a solid analysis of how morals change between gender and age demographics. To provide a more solid background it might be helpful to provider a copy of the Moral Foundations Questionnaire, the first and second version to fully understand the differences. In addition, it might be helpful to elaborate on the different domains care, fairness, proportionality, loyalty authority, and purity.

**********

6. PLOS authors have the option to publish the peer review history of their article (what does this mean?). If published, this will include your full peer review and any attached files.

If you choose “no”, your identity will remain anonymous but your review may still be made public.

**Do you want your identity to be public for this peer review?**  For information about this choice, including consent withdrawal, please see our Privacy Policy.

Reviewer #1: No

Reviewer #2: **Yes:**  Paula Beatty

**********

[NOTE: If reviewer comments were submitted as an attachment file, they will be attached to this email and accessible via the submission site. Please log into your account, locate the manuscript record, and check for the action link "View Attachments". If this link does not appear, there are no attachment files.]

To ensure your figures meet our technical requirements, please review our figure guidelines: https://journals.plos.org/plosone/s/figures

You may also use PLOS’s free figure tool, NAAS, to help you prepare publication quality figures: https://journals.plos.org/plosone/s/figures#loc-tools-for-figure-preparation.

NAAS will assess whether your figures meet our technical requirements by comparing each figure against our figure specifications.

# Author comment: Author response to Decision Letter 1

**DOI:** 10.1371/journal.pone.0352584.r002

28 Jan 2026

Dear Dr. Carrasco-Farré,

Thank you for the opportunity to revise our manuscript. We are grateful to you and the reviewers for the positive evaluation of our design and methodology, and for the constructive feedback regarding the theoretical framing and discussion of implications.

We have addressed all points raised. Specifically, we have:

1. Clarified the theoretical basis for our hypotheses regarding proportionality and effect sizes.

2. Expanded the discussion on the societal and political implications of these age/gender gaps.

3. Elaborated on the "maturational vs. societal" mechanisms in the Conclusion.

4. Provided detailed definitions of the six MFQ-2 domains and clarified the "distributional effects" terminology throughout the manuscript.

5. Added explicit text regarding cross-sectional design limitations.

Below is a detailed point-by-point response to the Academic Editor and Reviewers.

________________________________________

Response to the Academic Editor

Editor Point 1: Clarify and motivate the hypotheses more explicitly.

"As Reviewer 1 notes, it would be helpful to expand on the reasoning behind specific predictions, such as why proportionality was expected to show a male advantage, and why you anticipated age-related increases across all foundations with effect sizes in the .05–.25 range."

Response:

We agree that the specific predictions for Proportionality (a new foundation in MFQ-2) and the specific effect size estimates required stronger grounding in the introduction.

• Regarding Proportionality: We have added text explaining that proportionality aligns with "equity" or meritocratic allocation, a domain where men consistently score higher in evolutionary psychology and economic game literature, contrasting with the "equality" domain where women typically score higher.

• Regarding Effect Sizes: We have clarified that the range estimate (.05–.25) was derived directly from the meta-analytic baselines established by Castilla-Estévez & Blázquez-Rincón (2021) and the specific findings of Milojev et al. (2014).

Change to Manuscript:

Original text: " Drawing on these MFQ-2 findings and the broader MFQ-1 literature, we hypothesized..."

New text: "Drawing on MFQ-2 findings and the broader MFQ-1 literature, we hypothesized that our study would show age-related increases across all foundations (effect sizes ranging from .05 to .25), except possibly equality, consistent with the distribution of age effects reported in large-scale MFQ-1 studies (Castilla-Estévez & Blázquez-Rincón, 2021). Given that proportionality is part of the binding domain in MFQ-2, we expected its age association to be comparable in magnitude to the other binding foundations. Regarding gender, we predicted women would score higher on the individualizing foundations (care and equality) and purity, while men would show higher scores on the binding foundations (proportionality, loyalty, and authority), consistent with prior MFQ-1 results and with Atari et al. (2023) who reported a modest male advantage in MFQ-2 proportionality. We further expected a male advantage in proportionality because it aligns more closely with equality- or merit-based allocation; across economic-game paradigms, men tend to endorse merit more and women equality more (Capraro, 2020; Fehr et al., 2006)." (page 6, lines 119 - 130)

Editor Point 2: Elaborate the implications of the observed age and gender differences.

"Reviewer 1 also asks for more discussion of how these differences in moral foundations may translate into broader social and political outcomes... Deepening this part of the Discussion will help readers see the societal relevance of your findings."

Response:

We have expanded the Discussion to explicitly link these psychometric differences to real-world phenomena, specifically political polarization and intergenerational value conflict (e.g., "culture wars").

Change to Manuscript:

New text: "These divergences have tangible societal implications. The sharp age-related decline in Equality concerns, combined with the rise in Binding foundations, suggests a mechanism for the well-documented conservative shift associated with aging. Furthermore, the 0.78 SD gap in binding scores between the youngest and oldest cohorts provides a quantitative basis for current intergenerational conflicts ('culture wars'). Where younger cohorts may view the prioritization of tradition and authority as inherently oppressive, older cohorts may view the dismissal of these foundations as a failure of moral duty. Such foundation-level disconnects complicate cross-generational political consensus." (page 12, lines 251 - 258)

Editor Point 3: Develop the point about maturational versus societal causes.

"Please expand this argument: outline what kinds of maturational mechanisms you have in mind, what societal changes might be relevant, and whether you would expect these to operate similarly or differently in the US and UK contexts."

Response:

We have expanded the Conclusion to distinguish between Social Investment Theory (maturation) and secular liberalization (societal change). We also noted that given the shared "WEIRD" (Western, Educated, Industrialized, Rich, Democratic) status of the US and UK, we expect similar mechanisms in both, though the baseline levels might differ.

Change to Manuscript (Conclusion):

Original text: "Findings underscore the importance of considering both maturational... and societal... causes as drivers of changes in moral values."

New text: "These findings underscore the importance of considering both maturational and societal drivers. Maturational mechanisms likely include 'social investment' processes, where taking on adult roles (parenthood, employment) necessitates increased valuation of stability, authority, and loyalty. Simultaneously, societal drivers likely reflect the 'Flynn effect for morality' or broad secular liberalization, pushing cohort means toward higher Care and lower Purity. Given their shared cultural and economic structures, we expect these dual pressures to operate similarly across US and UK contexts, though their relative strength may vary with national factors such as religiosity and political culture." (page 13, lines 275 - 282)

Editor Point 4: Provide more background on the MFQ-1/MFQ-2 and the moral domains.

"Please elaborate briefly on the six moral domains... and clarify how MFQ-2 differs from MFQ-1... provide the MFQ-1 and MFQ-2 item sets as Supporting Information files..."

Response:

We have expanded the introduction to clearly define the six domains. We have also uploaded the full item sets for MFQ-1 and MFQ-2 as a Supporting Information file (S1_File_MFQ_Items.pdf and S2_File_MFQ -2_Items.pdf) as requested.

Change to Manuscript:

Original text: "The MFQ-2 differs from the MFQ-1 in two notable ways…"

New text: " Moral foundations theory (MFT; Atari et al., 2023; Graham et al., 2011; Haidt & Joseph, 2004) proposes that moral judgment is supported by multiple partially distinct concerns rather than a single moral principle. In this paper we focus on the six foundations assessed by the MFQ-2: care, equality, proportionality, loyalty, authority, and purity. Care captures concern for others’ suffering and harm prevention. Equality reflects egalitarian concern that people should be treated similarly and have similar outcomes. Proportionality reflects merit-based fairness - benefits and punishments should track effort, contribution, or responsibility (conceptually related to mutualism; Baumard et al., 2013; Lin & Bates, 2022). Loyalty concerns commitment to one’s group and aversion to betrayal. Authority concerns respect for legitimate hierarchy, tradition, and role-based duties. Purity concerns contamination and sanctity, including bodily and spiritual cleanliness and self-control. These foundations are often summarized into higher-order clusters in Western samples, with individualizing concerns (care, equality) emphasizing protection and equal regard for individuals, and binding concerns (loyalty, authority, purity, and proportionality) emphasizing group cohesion, order, and norm enforcement. (Atari et al., 2023; Graham et al., 2011).

Moral foundations are commonly measured with the MFQ-1 (Graham et al., 2011) and the updated MFQ-2 (Atari et al., 2023). The key conceptual difference is that MFQ-1 assesses five foundations, including a single fairness scale, whereas recently developed MFQ-2 assesses six foundations by splitting fairness into two separable constructs: equality and proportionality. In addition, MFQ-2 replaces the original MFQ-1 items with newly written or rephrased items, intended to improve clarity and content coverage and to better recover the expected factor structure in contemporary samples (Atari et al., 2023; Zakharin & Bates, 2023), addressing well-documented psychometric limitations of MFQ-1 in some datasets (e.g. Davies et al., 2014; Doğruyol et al., 2019; Iurino & Saucier, 2018; Kivikangas et al., 2017).The complete item sets for MFQ-1 and MFQ-2 are provided in the Supporting Information." (page 4, lines 67 - 74)

Editor Point 5: Clarify the “distributional effects” framing.

"The title and framing emphasise “distributional effects,” but the meaning of this term... could be described more explicitly."

Response:

We used "distributional effects" to refer specifically to the shape of the score distributions (skewness/kurtosis) and the identification of ceiling/floor effects, rather than variance heterogeneity. We have clarified this definition in the Introduction and Results to avoid ambiguity. We also changed the title to “Age, gender, and score distributions of moral foundations”

Change to Manuscript:

New text (Introduction): "... to describe mean differences in moral foundations by age and gender and the shape of MFQ-2 scores in the population—specifically examining distributional properties such as skew and ceiling/floor effects—and the changes exhibited with age." (page 3, lines 49 - 52)

New text (Results): "For each foundation (and the binding/individualizing composites), we summarized score distributions using the mean and SD and quantified distributional shape using skewness and kurtosis. We also inspected histograms to identify clustering near the minimum or maximum response options (floor/ceiling effects)" (page 9, lines 167 - 171)

Editor Point 6: Multiple comparisons and effect size interpretation.

"State how you addressed the issue of multiple testing... consider adding a short paragraph in the Discussion that contextualises the magnitude of the observed effects"

Response:

Given the large sample size (N > 2400 total) and the exploratory nature of the demographic mapping, we prioritized estimation of effect sizes (beta) over strict p-value thresholding, though we note that our key findings are significant at p < .001, robust to Bonferroni correction for the 12 primary tests (6 domains × 2 demographic variables).

Change to Manuscript (Results):

New text: "Given the number of comparisons across six moral foundations, two predictors (age, gender), and two samples, we prioritized the magnitude and consistency of standardized coefficients (β) over reliance on individual p-values. Nonetheless, we note that the majority of primary effects reported below survive a Bonferroni-corrected threshold of α = .05/12 = .004 per sample, and effects that replicated across both samples provide stronger evidence than any single test." (page 10, lines 204 - 209)

Editor Point 7: Age, cohort, and design limitations.

"Because the data are cross-sectional, age effects are potentially confounded with cohort and period effects... acknowledge this explicitly in the Limitations section"

Response:

We have reinforced the Limitations section to explicitly state the Age/Period/Cohort confound.

Change to Manuscript:

Original text: "Our cross-sectional design cannot disentangle age effects from cohort effects..."

New text: "The associations we report are between-person age differences and cannot be taken as evidence of within-person developmental (maturational) change. Age is confounded with birth cohort and historical period: older participants differ not only in age but also in the social and cultural context in which they were raised. Thus, the observed age-related increase in binding foundations (and decrease in equality) could reflect maturation, cohort-based socialization, or both. Longitudinal—ideally cohort-sequential—data are needed to separate age, cohort, and period influences." (page 13, lines 291 - 297)

________________________________________

Response to Reviewers

Reviewer #1

We thank Reviewer 1 for their assessment that the manuscript is "well-written and well-designed"15.

R1 Point 1: Explain hypotheses (Proportionality male advantage, effect sizes). 16

Response: As detailed in our response to the Editor (Point 1), we have revised the Introduction to explicitly ground the Proportionality hypothesis in literature suggesting male preferences for equity/meritocracy over equality and justified the effect size prediction based on the Castilla-Estévez meta-analysis.

R1 Point 2: Discuss repercussions/implications.

Response: We have expanded the Discussion to address political affiliations and intergenerational value conflict, as requested. (See response to Editor Point 2).

R1 Point 3: Expand on maturational vs. societal causes.

Response: We have elaborated on the Conclusion to contrast social investment mechanisms against secular shifts in values. (See response to Editor Point 3).

Reviewer #2

We thank Reviewer 2 for their comments regarding the solid analysis.

R2 Point 1: Provide a copy of the MFQ-1 and MFQ-2. 20

Response: We have created a Supporting Information files containing the items for the MFQ-1 and MFQ-2 to assist readers in understanding the instrument.

R2 Point 2: Elaborate on the different domains.

Response: We have revised the Introduction to provide distinct, conceptual definitions for Care, Equality, Proportionality, Loyalty, Authority, and Purity. (See response to Editor Point 4).

________________________________________

Specific Ethics Statement Update

Per Journal Requirement #222, we have updated the Methods section:

" The present study is a secondary analysis of previously collected data that are publicly available in de-identified form. The original data collection was approved by the Psychology Research Ethics Committee at the University of Edinburgh. Informed consent was obtained from all participants electronically prior to data collection. The current analyses used only anonymized, publicly shared data and therefore did not involve additional participant contact or collection of identifiable information."

Data Availability

Data remains available as per the original submission.

We believe these revisions significantly strengthen the manuscript and look forward to your decision.

Sincerely,

Michael Zakharin & Timothy C. Bates

Department of Psychology, University of Edinburgh

Attachment Submitted filename: 2 Response to reviewers.docx

# Peer review: Decision Letter 1

**DOI:** 10.1371/journal.pone.0352584.r003

**Authors:** Carlos Carrasco-Farré

11 Jun 2026

<p>Age, gender, and score distributions of moral foundations

PONE-D-24-60347R1

Dear Dr. Zakharin,

We’re pleased to inform you that your manuscript has been judged scientifically suitable for publication and will be formally accepted for publication once it meets all outstanding technical requirements.

Within one week, you’ll receive an e-mail detailing the required amendments. When these have been addressed, you’ll receive a formal acceptance letter and your manuscript will be scheduled for publication.

An invoice will be generated when your article is formally accepted. Please note, if your institution has a publishing partnership with PLOS and your article meets the relevant criteria, all or part of your publication costs will be covered. Please make sure your user information is up-to-date by logging into Editorial Manager at Editorial Manager® and clicking the ‘Update My Information' link at the top of the page. For questions related to billing, please contact billing support.

If your institution or institutions have a press office, please notify them about your upcoming paper to help maximize its impact. If they’ll be preparing press materials, please inform our press team as soon as possible -- no later than 48 hours after receiving the formal acceptance. Your manuscript will remain under strict press embargo until 2 pm Eastern Time on the date of publication. For more information, please contact onepress@plos.org.

Kind regards,

Carlos Carrasco-Farré

Academic Editor

PLOS One

Additional Editor Comments (optional):

Reviewers' comments:

Reviewer's Responses to Questions

**Comments to the Author**

1. If the authors have adequately addressed your comments raised in a previous round of review and you feel that this manuscript is now acceptable for publication, you may indicate that here to bypass the “Comments to the Author” section, enter your conflict of interest statement in the “Confidential to Editor” section, and submit your "Accept" recommendation.

Reviewer #2: (No Response)

**********

2. Is the manuscript technically sound, and do the data support the conclusions?

The manuscript must describe a technically sound piece of scientific research with data that supports the conclusions. Experiments must have been conducted rigorously, with appropriate controls, replication, and sample sizes. The conclusions must be drawn appropriately based on the data presented.

Reviewer #2: Yes

**********

3. Has the statistical analysis been performed appropriately and rigorously?

Reviewer #2: Yes

**********

4. Have the authors made all data underlying the findings in their manuscript fully available?

The PLOS Data policy requires authors to make all data underlying the findings described in their manuscript fully available without restriction, with rare exception (please refer to the Data Availability Statement in the manuscript PDF file). The data should be provided as part of the manuscript or its supporting information, or deposited to a public repository. For example, in addition to summary statistics, the data points behind means, medians and variance measures should be available. If there are restrictions on publicly sharing data—e.g. participant privacy or use of data from a third party—those must be specified.

Reviewer #2: Yes

**********

5. Is the manuscript presented in an intelligible fashion and written in standard English?

PLOS ONE does not copyedit accepted manuscripts, so the language in submitted articles must be clear, correct, and unambiguous. Any typographical or grammatical errors should be corrected at revision, so please note any specific errors here.

Reviewer #2: Yes

**********

6. Review Comments to the Author

Please use the space provided to explain your answers to the questions above. You may also include additional comments for the author, including concerns about dual publication, research ethics, or publication ethics. (Please upload your review as an attachment if it exceeds 20,000 characters)

Reviewer #2: Paula Beatty's Comments to Authors

Overall, this research about moral foundations is very well written and insightful. Reviewer 1 asks for more of a discussion on observed differences in moral foundations relates to broader sociopolitical outcomes. A more detailed analysis of sociopolitical might help the reader understand the overall significance of this study. Also, these points seem to be slightly contradictory: “Given their shared cultural and economic structures, we expect these dual pressures to operate similarly across US and UK contexts, though their relative strength may vary with national factors such as religiosity and political culture” in the Discussion section; and in the limitations section, “our focus on US and UK samples limits generalizability as cultures imbue moral values with different significance.”

Major Comments:

1. Expanding the definition for “culture wars” might help the reader understand why these results are still meaningful.

2. “Liberty” is removed in the revised version, even though liberty/oppression” are sometimes included in MFQ-2. It might be prudent to add liberty/oppression as a domain that is sometimes included in the proportionality domain since this is difference between MFQ-1 and MFQ-2.

**********

7. PLOS authors have the option to publish the peer review history of their article (what does this mean?). If published, this will include your full peer review and any attached files.

If you choose “no”, your identity will remain anonymous but your review may still be made public.

**Do you want your identity to be public for this peer review?**  For information about this choice, including consent withdrawal, please see our Privacy Policy.

Reviewer #2: **Yes:**  Paula Beatty

**********

Attachment Submitted filename: PONE-D-24-60347_R1.pdf

# Editor report: Acceptance letter

**DOI:** 10.1371/journal.pone.0352584.r004

**Authors:** Carlos Carrasco-Farré

PONE-D-24-60347R1

PLOS One

Dear Dr. Zakharin,

I'm pleased to inform you that your manuscript has been deemed suitable for publication in PLOS One. Congratulations! Your manuscript is now being handed over to our production team.

At this stage, our production department will prepare your paper for publication. This includes ensuring the following:

* All references, tables, and figures are properly cited

* All relevant supporting information is included in the manuscript submission,

* There are no issues that prevent the paper from being properly typeset

You will receive further instructions from the production team, including instructions on how to review your proof when it is ready. Please keep in mind that we are working through a large volume of accepted articles, so please give us a few days to review your paper and let you know the next and final steps.

Lastly, if your institution or institutions have a press office, please let them know about your upcoming paper now to help maximize its impact. If they'll be preparing press materials, please inform our press team within the next 48 hours. Your manuscript will remain under strict press embargo until 2 pm Eastern Time on the date of publication. For more information, please contact onepress@plos.org.

You will receive an invoice from PLOS for your publication fee after your manuscript has reached the completed accept phase. If you receive an email requesting payment before acceptance or for any other service, this may be a phishing scheme. Learn how to identify phishing emails and protect your accounts at https://explore.plos.org/phishing.

If we can help with anything else, please email us at customercare@plos.org.

Thank you for submitting your work to PLOS ONE and supporting open access.

Kind regards,

PLOS ONE Editorial Office Staff

on behalf of

Dr. Carlos Carrasco-Farré

Academic Editor

PLOS One
